# Electromigrated Gold Nanogap Tunnel Junction Arrays: Fabrication and Electrical Behavior in Liquid and Gaseous Media

**DOI:** 10.1021/acsami.4c03282

**Published:** 2024-07-02

**Authors:** Shyamprasad N. Raja, Saumey Jain, Javier Kipen, Joakim Jaldén, Göran Stemme, Anna Herland, Frank Niklaus

**Affiliations:** † Division of Micro and Nanosystems (MST), School of Electrical Engineering and Computer Science (EECS), KTH Royal Institute of Technology, SE-10044 Stockholm, Sweden; ‡ Division of Nanobiotechnology, SciLifeLab, Department of Protein Science, School of Engineering Sciences in Chemistry, Biotechnology and Health (CBH), KTH Royal Institute of Technology, SE-10044 Stockholm, Sweden; § Division of Information Science and Engineering (ISE), School of Electrical Engineering and Computer Science (EECS), KTH Royal Institute of Technology, SE-10044 Stockholm, Sweden; ∥ AIMES-Center for the Advancement of Integrated Medical and Engineering Sciences, Department of Neuroscience, Karolinska Institute, SE-17177 Solna, Sweden

**Keywords:** nanogap, electromigration, tunnel junction, single molecule sensing, nanofabrication

## Abstract

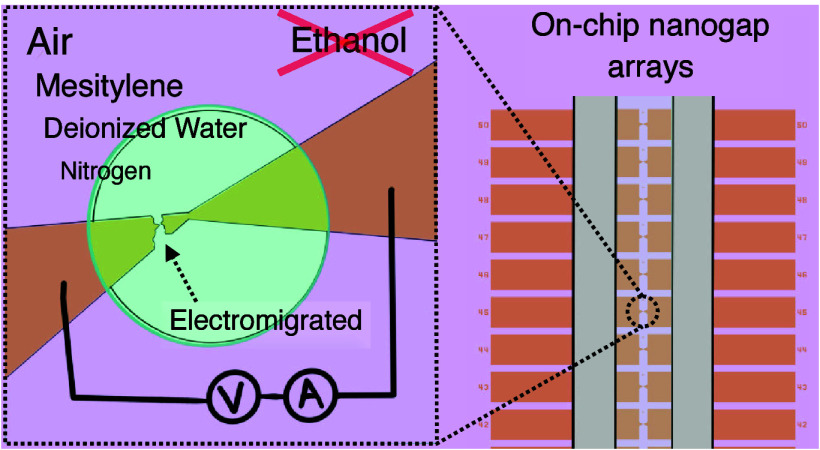

Tunnel junctions have been suggested as high-throughput electronic single molecule sensors in liquids with several seminal experiments conducted using break junctions with reconfigurable gaps. For practical single molecule sensing applications, arrays of on-chip integrated fixed-gap tunnel junctions that can be built into compact systems are preferable. Fabricating nanogaps by electromigration is one of the most promising approaches to realize on-chip integrated tunnel junction sensors. However, the electrical behavior of fixed-gap tunnel junctions immersed in liquid media has not been systematically studied to date, and the formation of electromigrated nanogap tunnel junctions in liquid media has not yet been demonstrated. In this work, we perform a comparative study of the formation and electrical behavior of arrays of gold nanogap tunnel junctions made by feedback-controlled electromigration immersed in various liquid and gaseous media (deionized water, mesitylene, ethanol, nitrogen, and air). We demonstrate that tunnel junctions can be obtained from microfabricated gold nanoconstrictions inside liquid media. Electromigration of junctions in air produces the highest yield (61–67%), electromigration in deionized water and mesitylene results in a lower yield than in air (44–48%), whereas electromigration in ethanol fails to produce viable tunnel junctions due to interfering electrochemical processes. We map out the stability of the conductance characteristics of the resulting tunnel junctions and identify medium-specific operational conditions that have an impact on the yield of forming stable junctions. Furthermore, we highlight the unique challenges associated with working with arrays of large numbers of tunnel junctions in batches. Our findings will inform future efforts to build single molecule sensors using on-chip integrated tunnel junctions.

## Introduction

Tunnel junctions typically are two terminal devices whose electronic transport characteristics are governed by an energy barrier across which quantum tunneling of electrons occurs.^1^ This is normally achieved by creating a physical nanoscale discontinuity in an otherwise continuous metallic conduction path. A specific type of tunnel junction formed as a nanogap across two tips made of noble metals, typically gold, narrowed to the atomic scale has been used as electrodes in the field of molecular electronics.^1−5^ The tunneling current in a nanogap tunnel junction is extremely sensitive to the nanogap width, the material of the electrode tips, the medium in the nanogap, and any molecules that transiently diffuse into the nanogap or transiently bond to and bridge across the electrode tips.^6^ By measuring the electron tunneling currents in nanogap tunnel junctions, immobilized as well as freely diffusing molecules present in the nanogap can be sensed at single entity resolution.^7−10^ Furthermore, by using specifically tailored recognition molecules anchored to the nanogap electrode tips which can reversibly bond to target molecules in solution,^11−14^ or by modifying the end groups of target molecules to bridge across the nanogap,^15^ the sensitivity of nanogap tunnel junctions can be further augmented. Proof-of-concept sensing of individual nucleotides, amino acids, short DNA and RNA strands, peptides, and proteins have all been demonstrated,^8,9,11,12,16^ raising the prospect of future high-bandwidth, high-throughput electronic biomolecular sensing and sequencing systems using tunnel junctions or, more generally, nanogap arrays as the foundation.^6,17−19^

Almost all seminal proof-of-concept biomolecular sensing results using nanogap tunnel junctions have been produced using break junction approaches.^7,8,11,12,15^ In these approaches, nanogap tunnel junctions are made using sub-nanoscale displacement control to deliberately break a gold nanowire to form the gap. Using these approaches, the nanogap can be reconfigured an arbitrary number of times, permitting a large number of experiments to be conducted using a single device by forming and breaking the junction on demand, producing robust statistically significant results associating tunneling current changes to molecules in the gap of the tunnel junction. However, these break junction approaches with reconfigurable gaps are not suitable for realizing large numbers of on-chip integrated nanogap sensors, as they require a means of producing individual mechanical deformation of each junction on the chip, which is extremely challenging to implement for miniaturized devices.^20^ To realize the vision of compact biomolecular sensing systems that can be closely integrated with electronics, the use of fixed-gap tunnel junctions with no moving parts that can be made by using wafer scale microfabrication processes is preferable.

Feedback-controlled electromigration,^21−25^ electrochemical deposition,^10,26,27^ crack lithography,^28−30^ spacer-layer-based gap formation,^14,31−34^ and focused electron/ion beam lithography^35−37^ are some techniques which have been proposed to realize fixed-gap tunnel junction devices. While these techniques can be used to fabricate on-chip integrated fixed-gap tunnel junctions, the size of the resulting nanogap cannot be precisely and deliberately changed after fabrication. This contrasts with the break junction approaches in which the nanogap can be mechanically reconfigured an arbitrary number of times after formation. To achieve electronic single molecule detection in nanogap tunnel junctions, it is desirable that the electrodes feature atomic scale tips where only a single gap dominates the electronic transport and the resulting tunneling current rather than a multitude of parallel conduction channels across the width of blunt electrodes. Among the above listed microfabrication approaches for on-chip integrated fixed-gap tunnel junctions, only electromigration, electrochemical deposition, and crack lithography have succeeded in producing tunnel junctions with atomic-scale tips.^10,30,38^

Despite the appeal of on-chip integrated fixed-gap tunnel junctions for large-scale sensing applications, their fabrication and application pose several challenges. The conductance of microfabricated tunnel junctions typically varies by several orders of magnitude, and the nanogap geometry can continue to change uncontrollably with or without the application of a bias voltage.^10,30,39^ Furthermore, once a fixed-gap junction stops functioning, it cannot be readily reformed. Therefore, any approach using fixed-gap tunnel junctions relies on the scalability of microfabrication to form very large numbers of junctions on a chip (from hundreds up to millions of devices) to obtain a reasonable number of tunnel junctions with the appropriate baseline conductance level.^40^ Moreover, the lifetime and, especially, the conductance stability of tunnel junctions under conditions appropriate for sensing applications are important parameters. Several studies from the field of molecular electronics have shown that gold nanogap tunnel junctions are inherently unstable at room temperature and exhibit significant conductance instabilities.^26,38,39,41^ It is unclear which parameters significantly affect the yield and stability of nanogap tunnel junctions, such as the materials of the nanogap electrodes, the geometry of the microfabricated junctions, the medium in which the junction is operated, the microfabrication process used, and any post-treatment applied to the junction, although this type of information is critically important to make further progress on nanogap tunnel junction sensor development. Very few studies using fixed-gap tunnel junctions have demonstrated proof-of-concept single biomolecule sensing,^10,14,42^ and only one of these studies which uses electrochemical deposition on carbon-coated glass pipettes (i.e., a process that is not compatible with wafer-scale microfabrication) broaches the issue of conductance instability in gold tunnel junctions.^10^ The aspects of yield and stability of on-chip integrated gold nanogap tunnel junctions during formation and operation in liquid media relevant for sensing applications have never been systematically investigated.

**Figure 1:**
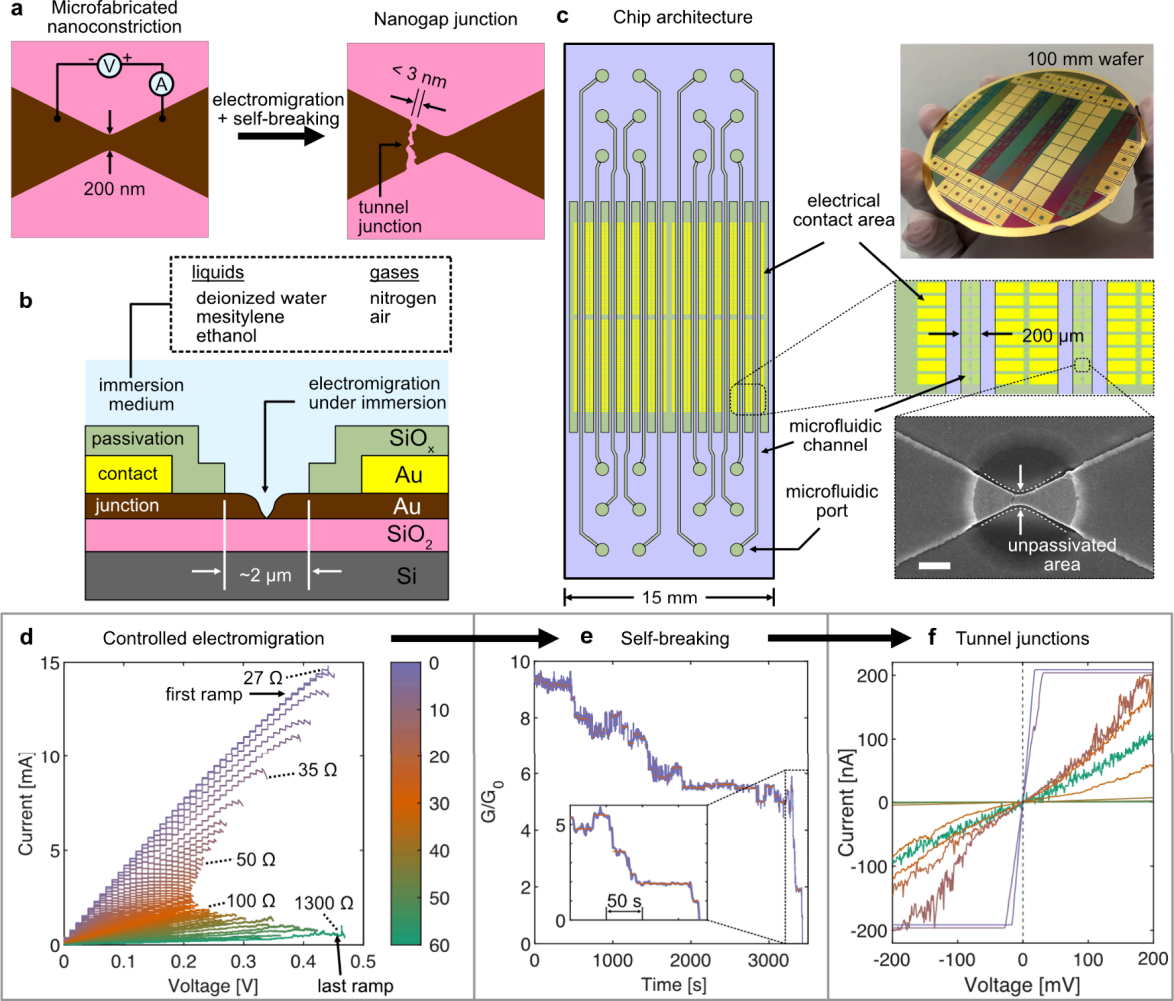
Gold nanogap tunnel junction and fluidic system architectures and nanogap fabrication and characterization approach used in this study. (a) Schematic of the top view of a microfabricated gold nanoconstriction device, which after electromigration and self-breaking produce a nanogap on the cathode side. A certain percentage of such devices form tunnel junctions. (b) Schematic of the cross section of a single nanoconstriction. Controlled electromigration and self-breaking of gold nanoconstrictions were performed under immersion in various media. Each nanoconstriction was in contact with the medium under study over a 2 μm diameter circular area which is free of the dielectric passivation layer (SiO_*x*_). (c) Schematic of the top view of a single 15 × 45 mm^2^ chip with a 12-channel OSTE flow cell (blue) bonded to the chip. Each microfluidic channel can be independently perfused and is aligned to an array of 100 individually addressable devices on the chip, as shown in the hierarchical magnified views. The chips were fabricated on 100 mm diameter wafers. The SEM image shows an ∼2 μm diameter circular unpassivated area of a nanoconstriction (scale bar 500 nm). (d) *I*–*V* characteristics of the controlled electromigration of a device in air. The resistance of this device was increased gradually over 59 ramps from 27 Ω to 1.3 kΩ (∼10 *G*_0_, where *G*_0_ is the conductance quantum (∼77 μS)). Note that time in the raw data was converted to the voltage shown in the *x*-axis using the known voltage ramp rate of 50 mV/s. The color bar shows the ramp number. (e) Self-breaking trace of a device after electromigration to ∼10 *G*_0_. The conductance spontaneously decreases in several discrete steps, eventually dropping to well below 1 *G*_0_. Inset shows the last 200 s of the recording where the conductance steps around 1 and 2 *G*_0_ can be observed. (f) *I*–*V* sweeps after self-breaking indicate whether a certain device has yielded a tunnel junction. Nine devices spanning a range of conductance from 0.4 nS to 11 μS are shown here.

Here, we take a significant step toward closing this gap in our understanding of on-chip integrated fixed-gap tunnel junctions using electromigrated gold tunnel junctions as the prototype. We are the first to study if and how the electromigration and self-breaking processes work in various liquid media (deionized water, mesitylene, ethanol) to produce nanogap tunnel junctions, and we systematically investigate the formation, stability, and electrical behavior of arrays of gold nanogap tunnel junctions in liquid and gaseous media (Figure 1). Starting from arrays of gold nanoconstrictions fabricated on wafer scale using standard microfabrication processes, we performed electromigration and self-breaking of the junctions directly in liquid, rather than only in air or in a vacuum as has been studied in the past (Figure 1a–d).^21,38,42,43^ The potential advantage of this strategy is that when nanogap tunnel junctions are formed directly in the medium in which they are to be operated as sensors, any stable atomic configurations found by electrode rearrangements during spontaneous self-breaking (Figure 1e) would not be destabilized by changing media afterward. We systematically analyzed the electronic transport characteristics of 332 nanogap junctions in various media after self-breaking to determine the yield of tunnel junction formation, which is found to be medium-dependent. We identify and classify different conductance instabilities in these nanogap tunnel junctions and identify operational conditions in different media that could affect this behavior. We also highlight the challenges that emerge when performing serial measurements on our arrays of tunnel junctions.

## Results and Discussion

To systematically investigate the formation and electrical behavior of arrays of gold nanogap tunnel junctions formed by feedback-controlled electromigration under immersion in various liquid and gaseous media (Figure 1a,b), we used a chip with 12 arrays of 100 nanoconstrictions each, where each array could be independently perfused with a fluid using a 12-channel microfluidic flow cell permanently bonded to the chip (Figure 1c). The gold nanoconstrictions were electromigrated in batches of approximately 50 devices in different media and allowed to self-break to yield tunnel junctions, which were then analyzed further (Figure 1d–f); the exact sample size and yield of all measurement batches in this work are summarized in Table S1. We fabricated the gold nanoconstrictions at wafer scale using anisotropic plasma etching of 27 nm thick gold films deposited by electron beam evaporation. A chromium layer with a nominal thickness of 2 nm was used as the adhesion layer to ensure the reliable adhesion of gold to the silicon dioxide (SiO_*x*_) surface of the silicon wafer (Figure 1b). Good adhesion of the gold was most critical during the wet etching of the SiO_*x*_ passivation layer deposited and patterned after fabrication of the nanoconstrictions. The passivation layer was necessary to minimize the wetted area of the gold electrodes during measurements in liquids to minimize parasitic current paths and the concomitant broadband electronic noise which would otherwise have been introduced to high bandwidth current measurements.^44−46^ Potential influences of the chromium adhesion layer on the electromigration process of the nanoconstrictions are discussed in Supplementary Note 1.

**Figure 2:**
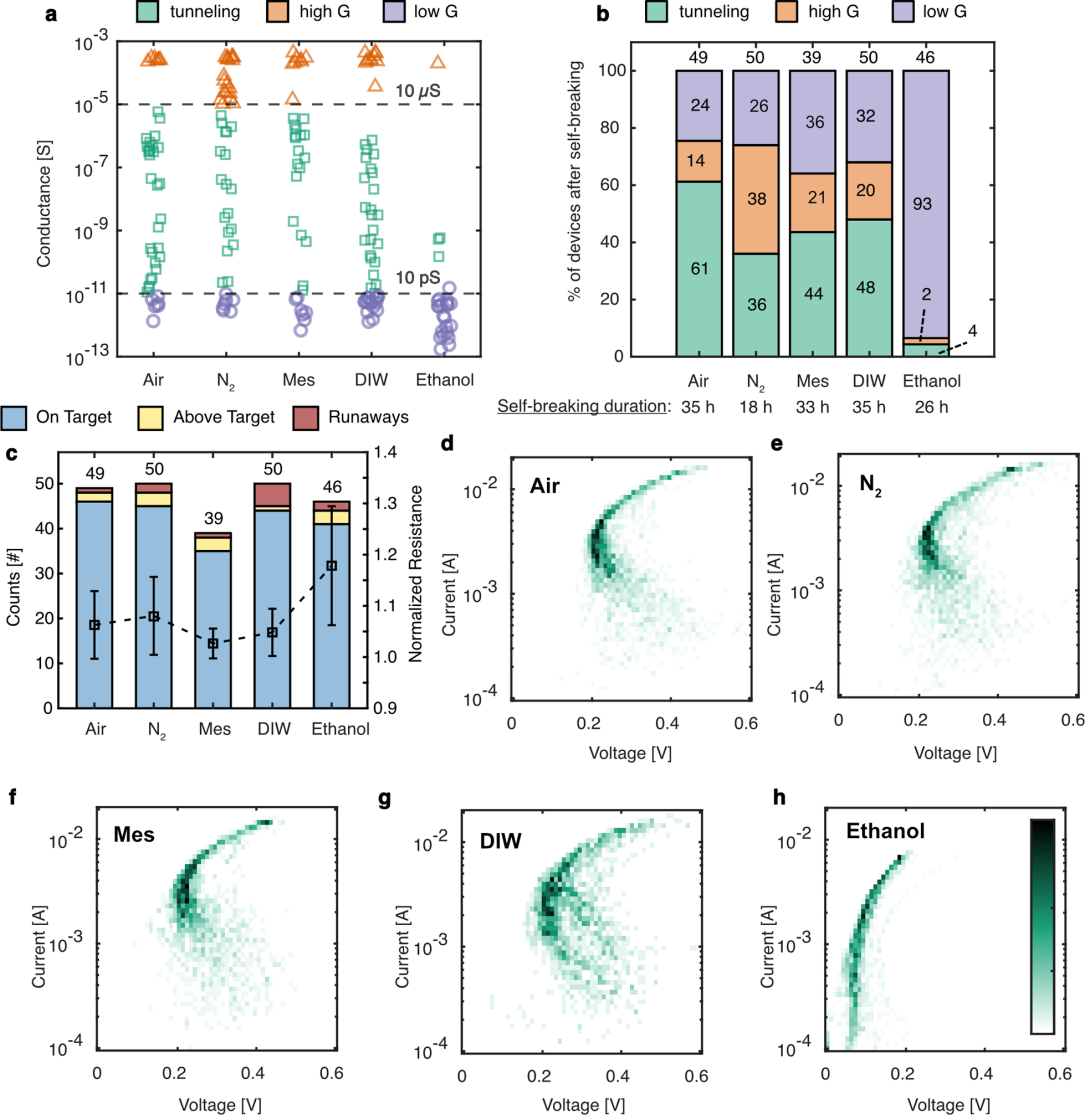
Summary statistics of controlled electromigration and self-breaking in various media, namely air, nitrogen (N_2_), mesitylene (Mes), deionized water (DIW), and ethanol. (a) The measured device conductances (*G*) after the self-breaking period has elapsed are spread over decades ranging from sub-pS to ∼100 μS. Based on their conductance, devices can be classified into three categories: (i) high *G* (*G* > 10 μS), (ii) tunneling (10 pS < *G* < 10 μS), and (iii) low *G* (*G* < 10 pS). Only the tunneling category represents tunnel junctions. The two horizontal dashed lines are guides to the eye to demarcate these three classes of junctions based on their conductance. (b) Bar graphs show the outcome of the self-breaking process after electromigration, grouping devices into the three categories based on their conductance as described in (a). The numbers inside each bar graph segment show the percentage of devices in each category. The yield of tunnel junctions is highest in air and least in nitrogen, with deionized water and mesitylene lying in between. Almost no tunnel junctions are produced in ethanol. Average duration of self-breaking at the time of measurement is also shown. (c) The bar graph shows a breakdown of the outcome of the feedback controlled electromigration process in each medium into three categories based on the device resistance (*R*) immediately after electromigration and before self-breaking: On Target (*R* < 10 kΩ), Above Target (10 kΩ < *R* < 100 kΩ), and Runaways (*R* > 100 kΩ). The total count indicated above each bar shows the actual number of samples in each medium where electromigration was carried out. The target resistance set for electromigration was either 1300 or 2600 Ω on an identical subset of devices in each medium. In all media electromigration runs controllably to the target resistance, with at least 89% of devices being on target. The average resistance after electromigration of devices which are classified as “On Target” normalized by the corresponding set target resistance is also shown (□, axis to the right). The error bars represent the mean average deviation of this statistic. (d–h) 2D histograms showing the collective current–voltage trajectory of controlled electromigration in different media. Each plot is constructed using the electromigration stopping trajectory (IV_Stop_) of all devices electromigrated in the respective medium. Note that the current axis is logarithmic, while the voltage axis is linear. The most remarkable observation is that the trajectory of electromigration is quite similar in all media, except ethanol. Color bar representing the counts in the 2D histogram shown in (h) is applicable to (d–g) as well and is a linear scale from white to black. The lower limit of the color bar is 0 in all cases.

We performed controlled electromigration of the gold nanoconstrictions (to 2.6 or 1.3 kΩ, equal to ∼5–10 *G*_0_ conductance level, chosen based on past studies of pure gold nanoconstrictions in air/vacuum;^38^ 1 *G*_0_ ∼ 77 μS is the quantum of conductance) and allowed self-breaking to occur (for >12 h) in batches immersed in five different media (deionized water (DIW), mesitylene (Mes), ethanol, nitrogen (N_2_), and air). The processes of feedback controlled electromigration and self-breaking are described in detail in the Methods section and in Supplementary Notes 2 and 3. The five media investigated in our study were chosen for the following reasons: nitrogen was studied to understand whether decreasing the water vapor and oxygen levels in air to trace amounts has a measurable impact on electromigrated devices; mesitylene was chosen because it is a stable nonpolar solvent used as a medium in molecular electronics studies;^41,47^ DIW was chosen for its polar protic nature and relevance for biomolecular sensing; ethanol was chosen because many interesting organic molecules are soluble in it, and it is used widely as a solvent to form self-assembled monolayers on gold. After electromigration and self-breaking, keeping the immersion medium unchanged, we measured the yield of tunnel junctions by using current–voltage (*I*–*V*) sweeps of all devices. The conductance (*G*) of each device was estimated by a linear fit performed over a voltage range of ±100 mV. The conductance of all devices grouped by the medium in which they were electromigrated and measured is shown in Figure 2a. In all media a vast majority of devices have self-broken to a level of conductance well below 1 *G*_0_, and the final conductances are spread over several decades ranging from <1 pS to >100 μS. Ethanol is the anomaly here, with almost all devices showing conductance less than 10 pS.

For further analysis of trends, we divided the devices into three categories (high *G*, tunneling, and low *G*) based on their conductance after self-breaking (Figure 2b): (i) high *G* (*G* > 10 μS), (ii) tunneling (10 pS < *G* < 10 μS), and (iii) low *G* (*G* < 10 pS). Of these categories only the devices sorted into the tunneling category are the primary target of this study due to the expectation of their suitability to act as sensors for small single molecules because: (i) they have self-broken to conductance well below 1 *G*_0_, which is one requirement for a gold tunnel junction,^1^ (ii) their conductance is high enough to produce a measurable current response at the voltage levels typically used in single molecule sensing (|*V*| ≤ 500 mV),^10,48^ and (iii) their physical gap sizes are in the 0.5–3 nm range (inferred from the voltage response assuming a barrier height of ∼1 eV).^30,48^ Devices in the high-*G* regime comprise mostly devices with conductance above 1 *G*_0_ (i.e., those whose conductance has barely decreased after electromigration). These high-*G* devices (14–38% depending on the medium) might have also yielded tunnel junctions under different conditions of electromigration and could be a future target to improve the yield of tunnel junctions. Devices in the low-*G* regime typically do not show a response to voltage bias below 200 mV and in many cases even up to 500 mV. Low-*G* devices might be useful for labeled nanogap sensing approaches such as recognition tunneling, where wider nanogaps can be used.^14,15,17^ The main focus of this study is on the devices classified as tunneling.

Considering all electromigrated devices, the obtained yield of tunneling devices is medium dependent and ranges from 36–61% of all devices evaluated (exact sample sizes are shown in Figure 2b and Table S1). Devices are most likely to self-break in air, whereas in N_2_ a greater proportion remain in the high-*G* regime (14% and 38%, respectively). The proportion of low-*G* devices in air and N_2_ (24% and 26%, respectively) are similar, as are devices in mesitylene and DIW (36% and 32%, respectively). Overall, the largest difference in yield of obtaining tunnel junctions occurs between air and N_2_ (gases), while mesitylene and DIW (liquids) lie in between and show quite similar spread of devices among the three categories. Ethanol, as already mentioned, is not a medium where tunnel junctions can be reliably formed by electromigration as performed in this study. Electromigration and self-breaking results from additional batches of devices in mesitylene, N_2_, and air produced similar figures for yield (34% and 40% in N_2_, 48% in mesitylene, and 67% in air for tunneling devices; full comparison in Figure S1) in these media compared to the first batches in the respective media, indicating a modicum of repeatability of these results. It is important to note that the precise conductance distribution of devices is a snapshot at that instant in time. After electromigration the conductance of an individual device continues to change, typically decrease, over time. Most of this change occurs in the first 12–24 h after electromigration (Supplementary Note 3, Figures S2 and S3). We found that when a self-breaking period longer than 9 h was used, the number of devices classified as tunneling in subsequent measurements performed up to 28 h later changes by less than 8% in all the media studied (Figure S1), and the overall spread of conductance of tunneling devices is similar. We also found that the conductance distribution of devices remains similar upon changing from mesitylene to nitrogen. This agrees with the expectation that in tunnel junctions not made in an ultrahigh-vacuum environment, gold work function lowering due to surface adsorbates makes conductance changes observed when changing between media to be relatively modest (Supplementary Note 5 and Figure S4).^10,36,48,49^

From these results, it is evident that in addition to gaseous media (air, N_2_) the feedback-controlled electromigration and self-breaking processes work under liquid immersion in mesitylene and deionized water but not in ethanol. There are several ways in which the immersion medium might impact these processes. First, it is the susceptibility of the chromium containing gold devices under the application of a voltage bias to any electrochemical reactions which might occur in liquid that is of primary concern. Second, the impact of parasitic current paths and the resulting electrohydrodynamic flows in liquid media might affect the heat sinking of the device. Third, once electromigration reduces the junction to a nanoscopic wire, the medium might stabilize the wires to delay or prevent the spontaneous self-breaking process. To elucidate if and how the electromigration and self-breaking processes are affected by liquid immersion, we analyzed the electromigration process in various media using electrical characterization as well as scanning electron microscopy (SEM).

We observe that electromigration could be performed controllably in all five media (even ethanol) studied (Figure 2c). When migrated to a target resistance of 1300 or 2600 Ω, 88–93% of devices have a final resistance after electromigration and before self-breaking of less than 10 kΩ (“On Target” category in Figure 2c). Note that a vast majority of these devices have a final resistance very close to the targeted resistance, showing that controlled electromigration is possible in all these media (Figure S5). The average normalized resistance after electromigration deviates by less than 10% from the targeted value in all media except ethanol, where both the average and the spread are the highest, at 1.18 ± 0.12 (Figure 2c). The control of electromigration seems to be marginally better in mesitylene and DIW compared with that in air and N_2_. Note that although each electromigration experimental batch consisted of 50 consecutive devices on a chip, electromigration was not carried out on a specific device if it did not show a low resistance ohmic *I*–*V* curve during a control measurement at the start. Therefore, the actual number of electromigrated samples might deviate from 50, as indicated in the relevant figure panels (Figure 2b,c).

The collective *I*–*V* trajectory of controlled electromigration of all devices studied in each medium is shown in Figure 2d–h as 2D histograms. Each plot is constructed by extracting the value of current and voltage at which electromigration starts to occur at each ramp for each device, i.e., the electromigration stopping trajectory (IV_Stop_; see Figure S6a for an example of a single device). The 2D histogram is then computed using a 10 mV resolution for the voltage axis and dividing the current range from 0.1 to 20 mA into 51 bins in the logarithmic scale. Therefore, these histograms of stopping trajectories of electromigration in various media are constructed using the cumulative behavior of 49, 50, 39, 50, and 46 devices each in air, N_2_, mesitylene, DIW, and ethanol, respectively. The initial 2–4 ramps where no measurable electromigration occurred are not included in these plots. Such initial ramps are observed in all media except ethanol.

From the 2D histogram plots of *I*–*V* trajectories, a clear distinction between ethanol and the rest of the media is evident. The normative behavior of controlled electromigration is for the *I*–*V* trajectory to describe a roughly hyperbolic shape whose spread increases as the resistance of the device increases, as exemplified by the data for electromigration in air (Figure 2d). Starting with a rather narrow spread at low resistance (high current values) the spread of data points gets progressively wider as the resistance increases (low current values) beyond the vertex of the hyperbola. While the shape of this curve can be understood, especially at the limit of *R* < 100 Ω by the critical power dissipation model,^24,50^ there is significant deviation from this model beyond the vertex of the curve; then devices are little more than sub-10 nm diameter nanowires (Supplementary Note 6). This is consistent with the current understanding that the fit values for series resistance and critical power, which are parameters of the model, might be very different later compared to at the start of the electromigration process due to the extremely local nature of electromigration once the junction has been reduced to a nanoscopic scale.^43,51^ While devices in air, N_2_, and mesitylene are almost indistinguishable in their overall response to electromigration in those media, devices in DIW show a clustering around two trajectories in the lower half of the curve (Figure 2g). Individual *I*–*V* trajectories of electromigration show a tendency of some devices (16 out of 50) in DIW to switch between the two curves rather than due to separate sets of devices which follow the inner or outer trajectory (Figure S7). Based on a similar observation in the electromigration trajectory of a pure gold junction in vacuum, Hoffmann-Vogel et al. proposed that such switching back and forth of the trajectory is possible if two parallel weak spots form during electromigration.^43^ Since one or the other weak spot is less resistive at the start of a certain electromigration ramp, more current flows through it, and the local nanoscale environment of this weak spot determines the critical power required for electromigration, and its resistance increases until the other hot spot becomes the dominant one in a subsequent ramp. An alternative explanation we considered for the switching back and forth of the critical power was the electrochemical oxidation of chromium in DIW. Chromium metal co-exists with chromium oxide in air at room temperature.^52^ If metallic chromium becomes newly exposed to DIW during electromigration, it might oxidize abruptly under the application of voltage bias. This would in turn cause an abrupt increase in resistance at a critical power lower than that of the previous ramp. Of the two explanations, since only the abrupt oxidation of chromium requires the presence of an oxidizing environment like DIW, it seems to be the more likely explanation for the splitting of the tail end of the electromigration trajectory in DIW observed in our study.

Nanoconstrictions electromigrated in ethanol show different characteristics compared to nanoconstrictions electromigrated in the other media from the earliest stages of the electromigration process (Figure 2h). Devices seem to migrate readily, and a naïve application of the critical power dissipation model indicates ∼10 times lower critical power required for electromigration, about 40 μW compared to 350–450 μW for electromigration in all other media. However, as discussed earlier, almost none of the devices that were electromigrated in ethanol formed usable tunnel junctions within the appropriate conductance range (Figure 2b). Particularly notable is the almost spontaneous monotonic increase in the resistance of nanoscopic gold wires in later stages of the electromigration process, as evidenced by stopping voltages that are well below 100 mV (Figure 2h). We hypothesize that this seeming eating away of gold atoms might be of electrochemical origin and operating in the nanoscale, as gold is not known to dissolve or corrode when simply immersed in ethanol. Gold is known to oxidize under the application of a positive voltage bias in ethanol even in the presence of trace amounts of water, and the gold oxide can be reduced again by lowering the bias.^53^ Further, gold oxide can be stripped in ethanol even without the application of any voltage bias, simply by immersion.^54^ During electromigration in ethanol, we are in effect ramping between 0 V and a positive voltage bias up to ∼250 mV and back, and we thereby create conditions very similar to those required to perform electrochemical redox processes in ethanol. However, do such redox processes typically result in the etching away of gold in ethanol? Using scanning tunneling microscopy, a previous study has shown that the morphology of a gold surface which has undergone UVO oxidation and ethanol immersion is different from that observed on a freshly evaporated gold surface,^55^ showing numerous depressions that are one to two atoms deep and a few nm to tens of nm in size. The authors argue that these depressions result from the removal of gold atoms from the surface, rather than simple atom rearrangement. If this is the case, then it could explain why in our study under conditions of electromigration the resistance of atomic scale wires of gold in ethanol could not be increased controllably due to the removal of gold atoms during reduction of gold oxide after the activation of the feedback loop during electromigration. As discussed later, *I*–*V* characterization of tunnel junctions in DIW seems to indicate that in DIW a similar reduction might only be triggered at bias voltages of <−300 mV. Here we never go to negative voltages during electromigration, and so the process worked well in DIW.

**Figure 3:**
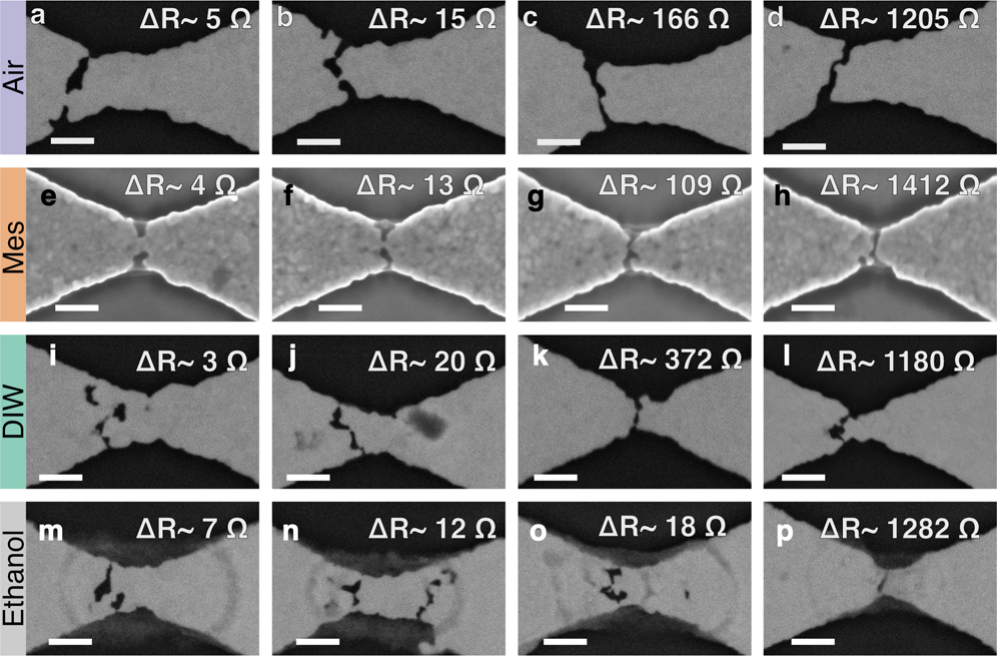
Progression of controlled electromigration in air and under liquid immersion: (a–d) in air, (e–h) in mesitylene, (i–l) in deionized water, and (m–p) in ethanol. In each image series, the targeted resistance after electromigration increases from left to right. This is shown as a resistance change (Δ*R*) from the starting resistance value (typically 25–30 Ω) of each device in the respective images. The SEM images of samples electromigrated in mesitylene were acquired using a secondary electron sensitive detector, whereas the images of samples electromigrated in other media were acquired using a backscattered electron sensitive detector, which is the reason for the contrast difference between the mesitylene series and the rest. All images were collected after electromigration was completed, and the chips were blow-dried. All scale bars are 200 nm.

To understand whether there are morphological differences in devices electromigrated in liquids compared with those in a gaseous medium, we performed SEM imaging of the junctions after controlled electromigration to a series of different target resistances under immersion. A selection of four SEM images each from devices electromigrated to different resistance targets in air (Figure 3a–d), mesitylene (Figure 3e–h), DIW (Figure 3i–l), and ethanol (Figure 3m–o) are shown in Figure 3. The SEM images of devices electromigrated in mesitylene and DIW show that the structure during electromigration changes in a manner very similar to that during electromigration in air. The formation of a nanogap occurs by the opening of a slit across the width of the nanoconstriction slightly off center toward the cathode side (toward the left from center in all panels of Figure 3). However, the cathodic offset is less pronounced in the samples in mesitylene compared to that in air. This is due to more effective heat sinking as we explain next. To facilitate SEM imaging after electromigration in liquid media, we used a different device geometry at a distance >2 μm away from the nanoconstriction (Supplementary Note 7 and Figure S8). This made the heat sinking of the nanoconstriction more effective and decreased the average cathodic offset of the slit formed after electromigration in these devices. Notably, in mesitylene we observed several instances where the offset of the slit toward the cathode was much lower than in DIW, likely due to the higher boiling point of mesitylene (164.7 °C) compared to DIW which enhances the heat sinking of the nanoconstriction (Figure 3e).^50,56^ We present further evidence of the link between the effectiveness of heat sinking of a nanoconstriction and the cathodic offset of the slit formed by electromigration in the Supporting Information (Supplementary Note 8, Figures S9 and S10). Despite these differences in the cathodic offset, consistent with the electromigration trajectories overall being similar in DIW and mesitylene compared to those in air (Figure 2d–g), the results from imaging agree with the understanding that no other phenomena seem to interfere with the process of electromigration in these liquid media. Again, for devices electromigrated in ethanol (Figure 3m–p) we observe something different. In addition to a slit toward the cathode side, several devices show a tendency to produce a secondary slit that is offset toward the anode side (toward the right from center in all panels of Figure 3). Also different from the slits on the cathode side, these anodic openings seem to nucleate and grow from the interior rather than the edges of the device (Figure 3n,o). However, since not all devices show such a secondary slit, this does not explain why devices in ethanol behave so differently under electromigration. The SEM images show that electromigration still occurs in ethanol and produces a cathodic slit like in the other media, although the resulting nanowires do not survive being electrically probed, as is evident from our electrical characterization results presented before. In addition, we also observe faint rings around the nanoconstriction after electromigration in ethanol (Figure 3m–p), reminiscent of the coffee-ring effect observed upon drying of droplets.^57^ These rings probably formed due to the local evaporation of ethanol during electromigration in the vicinity of the nanoconstrictions. The poor secondary electron contrast and dark backscattering contrast of these rings indicate it is likely carbonaceous rather than metallic deposition (Figure S10c,f). A more detailed SEM image series of devices electromigrated in air is presented in Figure S6 along with a description of the similarity of the morphological progress of electromigration of our gold devices with a chromium adhesion layer in air compared to previous reports of electromigration of pure gold junctions (Supplementary Note 6), which indicates that any partial intermixing of the chromium adhesion layer and gold did not create a noticeable difference to the electromigration process.^58^

**Figure 4:**
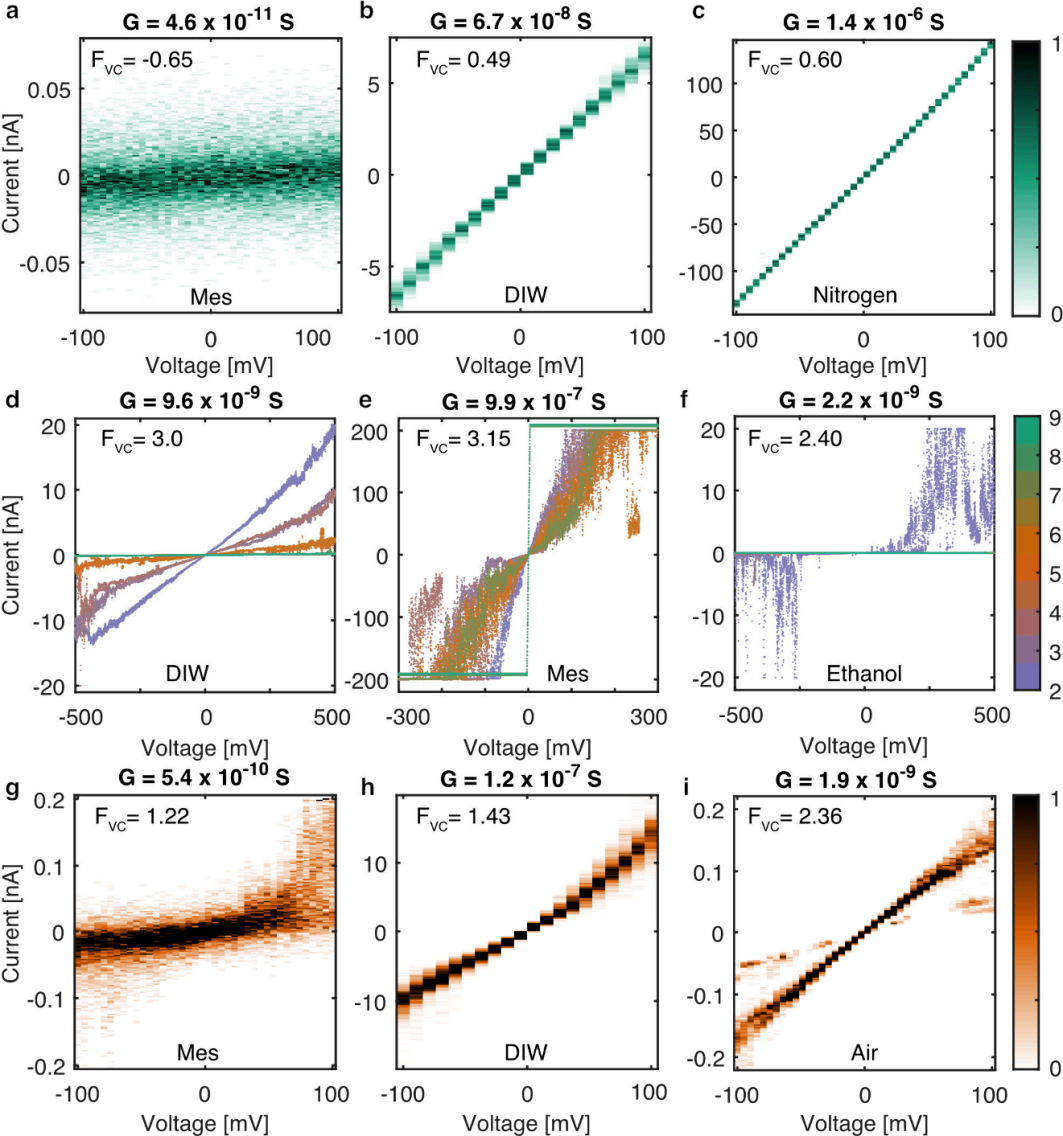
Current–voltage (*I*–*V*) characteristics of a selection of tunnel junctions displaying stable (a–c), reconfiguration (d–f), and fluttering (g–i) conductance characteristics spanning a broad range of conductance (*G*) and in various media. (a–c) *I*–*V* histograms show stable conductance with minimal current spread at any voltage. (d–f) *I*–*V* sweeps colored by the sweep number show significant and abrupt decrease or increase in conductance from one sweep to the next, a type of conductance instability we call reconfiguration. (g–i) *I*–*V* histograms show unstable conductance of a type we call fluttering with moderate to high levels of current spread at any voltage. For parts a–c and g–i, the color bar represents normalized counts of the *I*–*V* histogram. For parts d–f, the color bar represents the integer number of the *I*–*V* sweep. Sweeps 1 and 10 are not shown as they did not span the entire voltage range plotted. *F*_vc_ is a parametrized measure of the stability of a tunnel junction as inferred from its *I*–*V* characteristics and is described in Methods and in Figure 7.

**Figure 5:**
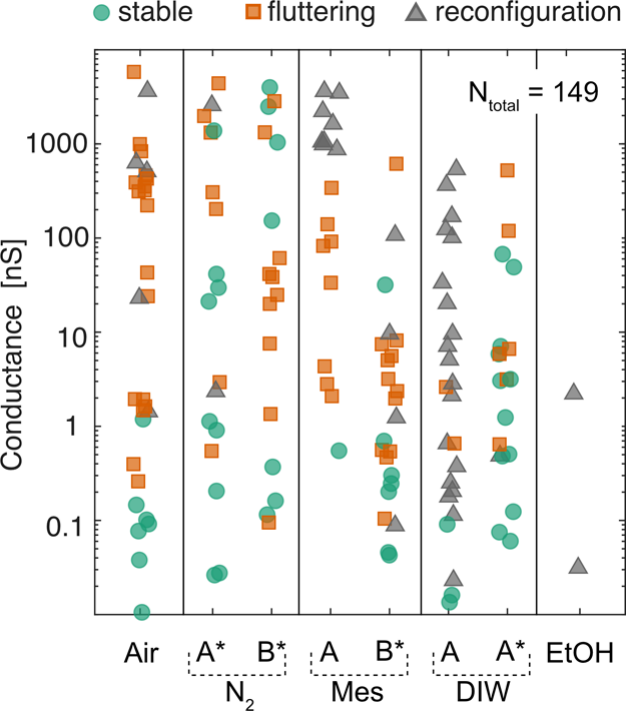
Classifying conductance stability characteristics of tunnel junctions. Plot showing the conductance of different tunnel junctions versus their stability classification in various media. Each tunnel junction was classified as stable, reconfiguration, or fluttering based on a visual assessment of its *I*–*V* characteristics. Each column represents a data set from a particular medium. The four data sets marked with an ∗ in the label were collected by biasing to a maximum of ±100 mV, whereas the rest were biased to ±500 mV. Mes-A, Mes-B* as well as N2-A*, N2-B* are independent batches of measurements. The two DIW data sets are not independent: DIW-A* was a second measurement to ±100 mV performed after 12 h on the same set of devices measured previously to 500 mV in DIW-A. We see that reconfiguration is absent in DIW-A*. Overall, we observe that stable conductance characteristics are more likely in devices which are less conductive (<∼10 nS). A total of 149 tunnel junctions were analyzed and classified across all media.

For use as molecular sensors, the conductance of nanogap tunnel junctions should be stable over a time period of at least several seconds and ideally several minutes, in the absence of deliberately added molecules so that transient changes, typically increases in conductance, can be attributed to the molecules interacting with the tunnel junction. Therefore, we investigated the conductance stability of our nanogap tunnel junctions in various media (i.e., only considering devices classified as tunneling as described earlier; also see Figure 2a). We performed ten *I*–*V* sweeps (acquired consecutively after going in contact once, at a scan rate of 100 mV/s) on all junctions and determined whether the conductance was stable or unstable. A device was classified as stable if for any voltage bias in the sweep the current histogram has a single peak (at the mean), and the spread is comparable to the noise floor for that current magnitude (Figure 4a–c). Among unstable devices, two main types of conductance instability were observed, which we term reconfiguration (Figure 4d–f) and fluttering (Figure 4g–i), discussed and illustrated further below. Further examples of *I*–*V* sweeps showing the typical S-shaped *I*–*V* characteristics expected for tunnel junctions are shown in Figure S11. The result of manual stability classification of *I*–*V* sweeps as stable, reconfiguration, or fluttering of all tunnel junctions analyzed in different media is shown in Figure 5.

The first kind of conductance instability in our tunnel junctions, reconfiguration, takes the form of a systematic change in the *I*–*V* characteristics of a device from one *I*–*V* sweep to the next (Figure 4d–f). Reconfiguration typically initiates abruptly above a certain applied voltage bias and is a useful indicator to determine the range of voltage levels within which the tunnel junctions made in this study can operate without being destabilized by the applied voltage in a certain medium. Reconfiguration in air and in mesitylene in particular was predominantly observed in tunneling devices with *G* > 100 nS when a bias exceeding ∼250 mV was applied and typically resulted in a significant decrease in conductance (Figure S12a), presumably as the tunneling gap widens in response to the high electric field as gold atoms at the tips reconfigure.^47,59^ Gold atoms on gold and dielectric surfaces at room temperature are known to have a high mobility, and this is the process which drives the spontaneous self-breaking process after electromigration to the ballistic regime.^38,60^ Even after a tunneling gap opens, this gap continues to widen over a period of hours to months as TEM studies have shown.^61^ Such widening can also be triggered by the application of increasingly larger voltages (in the range for 100–500 mV for devices with conductances between 100 μS and 10 nS) to a tunnel junction which causes uncontrollable order of magnitude decreases in conductance, indicating atomic scale rearrangements due to a moderate applied voltage,^24^ in agreement with our findings. In our study occasional counterexamples were also found, where instead a significant increase in conductance was observed (Figure 4e and Figure S12b). By avoiding the application of voltage bias more than ∼250 mV to devices whose conductance exceeded 100 nS, such large destabilization of devices was largely avoided, as seen from the three independent sets of measurements in N_2_ and mesitylene where *I*–*V* sweeps were only conducted to ±100 mV (see ∗ marked columns in Figure 5). Any reconfiguration observed subsequently only resulted in small changes in conductance (Figure S13).

In DIW, we observed a different manifestation of reconfiguration, which appears to be electrochemical in origin. While devices withstood bias up to 500 mV at positive polarity, with the *I*–*V* characteristic remaining ohmic at the start, a sudden onset of another electrically driven process emerges at negative polarity above 300 mV which resulted in an abrupt decrease of the device conductance (Figure 4d; further examples in Figure S14a–d). This pattern repeated itself over one or more cycles, eventually producing devices whose conductance was orders of magnitude lower than at the start of the *I*–*V* sweeps. We hypothesize that this reconfiguration might be related to the electrochemical reduction of chromium oxide in the vicinity of the gold tunnel junction. Chromium in the vicinity of the tunneling gap formed after self-breaking is likely to have oxidized to Cr_2_O_3_ during the prolonged immersion in DIW.^62^ The abrupt conductance decrease observed at *V* < −300 mV could then be due to mechanical displacements of the gold junction caused by the electrochemical reduction of Cr^3+^ to Cr^2+^ (the standard reduction potential is ∼−0.407,^63^ indicating that the reduction of chromium is possible at moderately low applied negative bias). If the measured current were dominated by tunneling current across a gold junction and the physical changes due to the reduction of Cr^3+^ in the vicinity of the junction caused atomic scale displacements of the cathode with each *I*–*V* sweep, it is probable that the tunnel junction would show abrupt changes in conductance above a certain applied negative voltage bias yet remain active afterward, since the gold is still intact albeit displaced. Devices which remained responsive to bias after these *I*–*V* sweeps when measured again to 100 mV did not show this type of systematic reconfiguration, showing that these electromigrated tunnel junctions can operate in DIW if lower voltage biases which do not trigger this electrochemical process were applied (Figure 4b and Figure S14e–h). Interestingly 12 out of 19 of the surviving devices also showed stable conductance characteristics afterward (see DIW-A* column in Figure 5 and Figure S15), indicating the possibility of using electrical stressing in DIW as a means of forcing a tunnel junction to find a more stable configuration.

**Figure 6:**
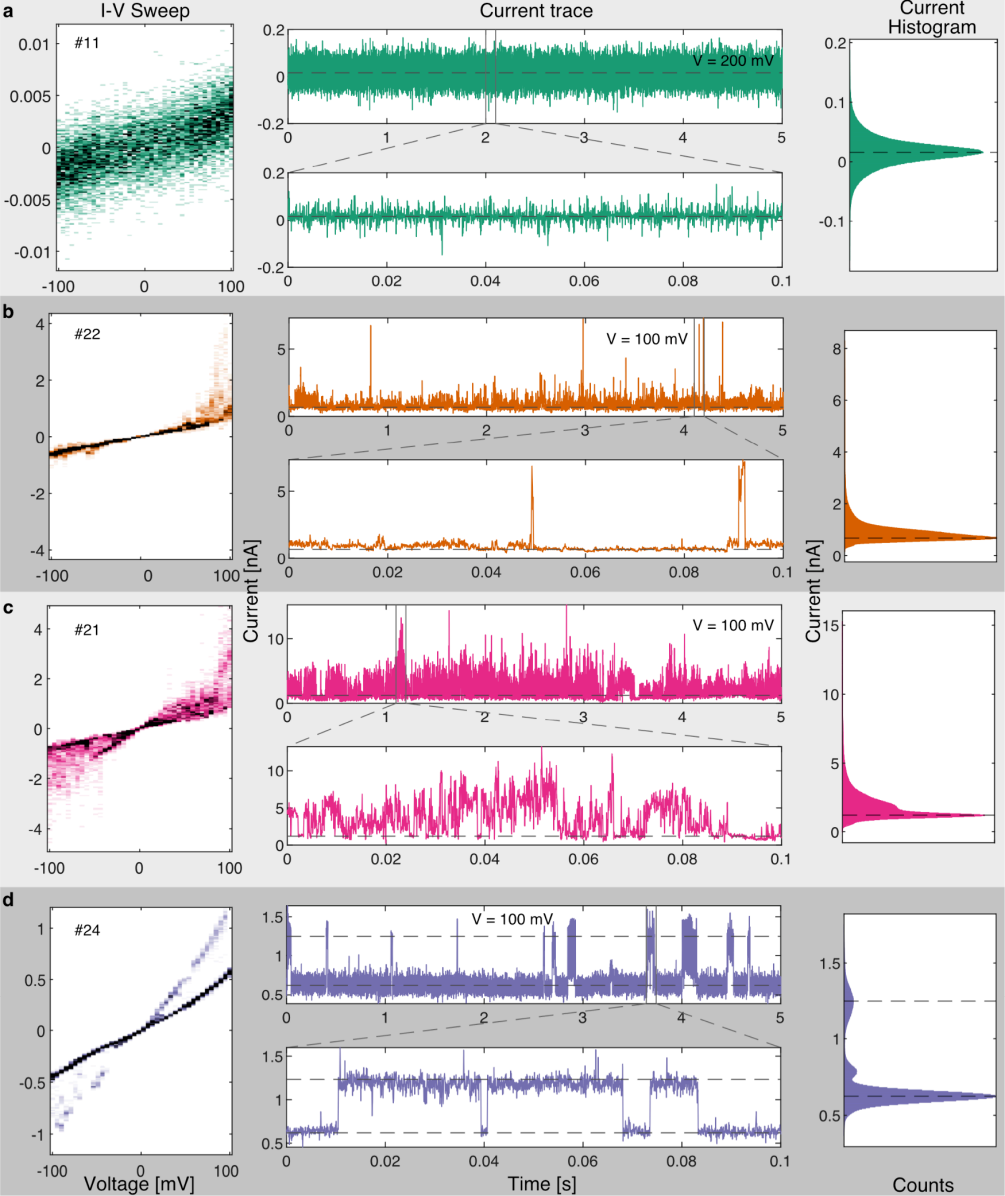
*I*–*V* sweep, current trace at fixed voltage bias, and current histogram of a selection of four tunnel junctions with different types of conductance characteristics in mesitylene. For each device, the *I*–*V* sweep (1.25 kHz, unfiltered) was acquired immediately before the current trace (50 kHz, low-pass filter at 10 kHz). Representative 5 s and 100 ms current traces are shown for each device in the upper and lower panels, respectively. The “all points” current histograms for the 5 s traces are shown on the right. The dashed lines in the current traces and current histograms represent characteristic peak positions identified from the histograms. (a) A stable device with a featureless current trace; (b) a fluttering device with distinct short intermittent current spikes; (c) a highly unstable fluttering device with constantly meandering current levels; and (d) a fluttering device with three distinct conductance states. Note that the numbers in the left panel (#11, etc.) indicate the device number in Figure 8a to which each row here corresponds to.

In the tunnel junctions with the second type of conductance instability, fluttering, *I*–*V* sweeps showed a continuous nonsystematic conductance fluctuation spread across a wide range of bias levels. Many tunnel junctions with conductance above 1 nS in all the media studied show this type of instability to varying degrees of severity (Figure 5, Figure S15, and Table S1). By comparing the *I*–*V* sweeps and current traces of stable (Figure 6a) and fluttering devices (Figure 6b–d), the manifestation of the transient current instabilities at fixed bias in the current traces can be observed as a larger spread in the *I*–*V* sweeps. Three examples of devices which show medium, high, and discrete conductance fluctuations in mesitylene are shown in parts b, c, and d of Figure 6, respectively. Fluttering is undesirable in the absence of molecules because it produces a current trace at fixed bias which can be very similar to transient current spikes over a relatively stable baseline level. This is problematic because such current spikes are also expected to occur when deliberately added molecules transiently diffuse into the tunneling gap or when they form transient molecular bridges.

**Figure 7:**
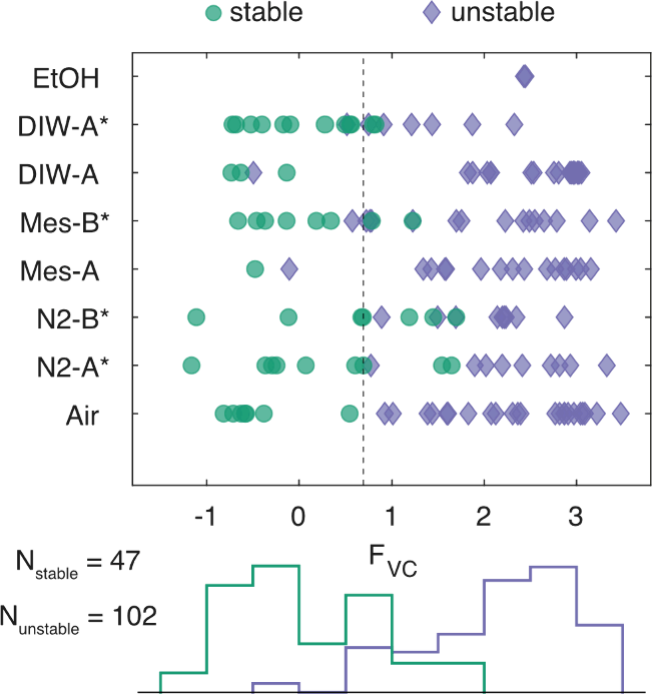
Parametric classification of conductance stability. A parametric feature *F*_vc_ calculated from the *I*–*V* characteristics of each tunnel junction can replicate the manual task of classifying a device as stable or unstable to an accuracy of 91% using a threshold value of *F*_vc_ = 0.69 (dashed line) for the 149 tunneling devices analyzed. Stable devices are distributed to the left of this threshold, and unstable devices are distributed to the right. This parameter could facilitate automation of the classification task to identify viable and stable sensors. The lower panel shows a normalized histogram of device counts for the two categories (stable, unstable) vs *F*_vc_. Note that the reconfiguration and fluttering categories in Figure 5 are aggregated to a single unstable category for this analysis.

While low levels of conductance fluttering, which produce a slight instability in the baseline conductance level, might be tolerated while sensing molecules that can produce much larger current spikes, it would be useful if we could quantify the level of fluttering so as to define an acceptable level while screening a batch of devices. This ability would also facilitate the automating of the classification of conductance stability. After an extensive analysis of *I*–*V* 2D histogram characteristics of 149 tunnel junctions using the manual labeling described earlier as the basis, we have identified an accurate parameter, *F*_vc_ (see the Methods section), for quantifying the stability of a tunnel junction. *F*_vc_ gauges the relative current variance due to the device instability, which is considered additive to the baseline noise because they are independent processes. Even though it depends on the background noise, *F*_vc_ has a greater accuracy in determining if a device is stable compared to the other tested parameters when considering data from eight data sets which include all the media studied (see the Methods section and Supplementary Note 9). In Figure 7, we present the distribution of *F*_vc_ for all data sets, demonstrating its capability in distinguishing stable and unstable devices. We found a 91% match between the parametric stability classification and the manual labeling for this data set using a threshold value of *F*_vc_ = 0.69 to set the boundary between stable and unstable devices. Devices with *F*_vc_ significantly to the right of the threshold exhibit high instability and cannot be used as sensors. Conversely, devices positioned far to the left of the threshold emerge as prime candidates for tunnel junction sensors due to their stable conductance characteristics. Devices near the threshold display transient flutter, which might not render them unusable as sensors, as the incidence of high-amplitude conductance excursion is relatively low.

Although a certain level of instability of gold tunnel junctions is expected,^26,41^ previous studies using various fabrication methods have had success in creating stable gold tunnel junctions at least for a time period long enough to use them as label-free single molecule sensors, typically working with one device at a time.^7,8,10,42^ The typical use case of a system like that in the present study, while working with arrays of tunnel junctions, is to perform control measurements of a batch of tunnel junctions in a blank medium, identify a set of stable devices, perfuse molecules, and then perform sensing experiments. To follow this strategy, we needed to determine whether devices would retain their conductance characteristics over multiple batches of measurements in the same medium. Therefore, we measured a set of 48 electromigrated devices in mesitylene three times in batches to determine whether devices retained their conductance value, conductance classification (tunneling, high *G*, or low *G*), and stability classification (stable or unstable (reconfiguration and fluttering are aggregated)) over this measurement series.

**Figure 8:**
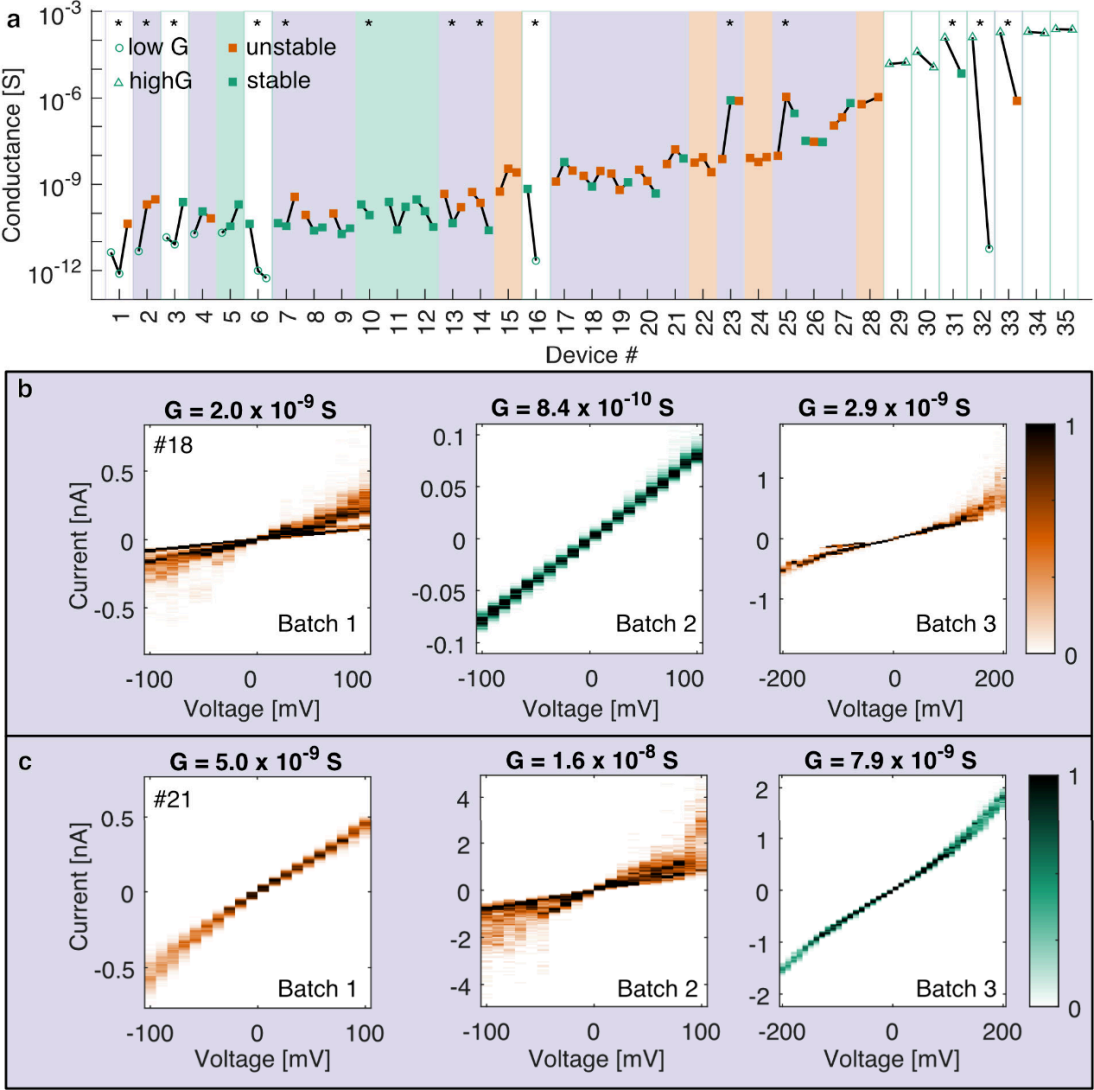
Results of a series of three measurements in batches of tunnel junctions in mesitylene. (a) Conductance of 35 devices measured in three batches, sorted from left to right in ascending order of conductance in the first measurement of the series. Different measurements of a specific device are arranged consecutively from left to right and connected by a solid black line. The marker type indicates the conductance category of a device (low *G*, tunneling, or high *G*), and for tunneling devices, the marker color indicates whether that particular *I*–*V* characteristic was stable or unstable (reconfiguration or fluttering). The column color of a device indicates whether its conductance remains always stable (green), always unstable (orange), or switches (blue) from stable to unstable or vice versa at least once during the measurement series. Eleven devices are not shaded at all because they were tunneling less than once during the series and cannot be used to infer conductance stability trends. All devices showing *a* > 10× conductance change during the series are marked with an ∗. (b) *I*–*V* histogram of device #18 and (c) device #21 showing transitions in the conductance characteristics from fluttering (orange) to stable (green) or stable to fluttering in the same tunnel junction.

In Figure 8a the results of 35 of these devices are shown, leaving out 15 devices that were low *G* in all three measurements of the series (see Supplementary Note 10 for a graphical summary of this experiment and the key results). Twenty-four devices (shaded columns in Figure 8a) were in the tunneling range at least twice during the measurement series and provided useful information regarding conductance stability. Of these, four were always stable, four were always unstable, and 16 change from stable to unstable or vice versa at least once during the measurement series. *I*–*V* histograms of two such devices whose conductance characteristics change from unstable to stable and vice versa during the measurement series are shown in Figures 8b and 8c. Further, seven of these devices show a conductance change greater than 10× during the series (all such devices are marked with an ∗ in Figure 8a), of which four show an increase and three a decrease in conductance. None of the four always stable devices ever have a conductance greater than 1 nS. However, 10 devices with conductance higher than 1 nS are stable in one or more individual measurements. It is this changeability of the conductance stability classification of devices in the ideal conductance range (>1 nS) to achieve high sensitivity to single molecules which is challenging to deal with when control measurements and molecule measurements are performed in batches, as these changes are difficult to predict.

We now discuss the possible causes of the observed conductance instabilities in our study. Charge traps in the gate dielectrics are known to cause random telegraph noise (RTN) in nanoscale electronic devices, and such traps can even be created due to the application of electric fields of the order of ∼0.15 V/nm in SiO_2_.^64^ RTN can also be caused by intrinsic or electric-field-induced defects in metal oxides, an effect used deliberately for the creation of memristor devices.^65^ Therefore, in our substrate-supported tunnel junctions formed by electromigration, potential intrinsic sources of conductance instabilities are charge traps in the dielectric substrate below the tunnel junction or in the chromium oxide in the vicinity of the gold tunnel junction. Two main causes have previously been discussed to understand conductance fluctuations in suspended tunnel junctions made using break junction approaches: (1) the forming, breaking, and reconfiguration of molecular bridges^30,59^ and (2) the rearrangement of atoms of the electrode tips around the tunneling gap.^26,41^ The formation of molecular bridges has in particular been attributed to switching between two distinct states whose conductance differs by several multiples (>2×, similar to in Figure 6d) or even orders of magnitude, whereas similar RTN switching observed in pure solvent has been reported to be a rare occurrence and of much lower magnitude, ∼*O*(1).^41^ While rearrangement of atoms of the electrode tips could explain abrupt and persistent conductance changes, they are usually not considered as a plausible cause for large amplitude conductance fluctuations at room temperature.^26,41^ Transient current spikes, such as those observed in our fluttering devices (Figure 6b,c), are expected when any entity capable of changing the tunneling barrier diffuses into the tunneling gap and diffuses out again. We have encountered such transient instabilities in devices in all the bare media studied, with mesitylene performing much worse than nitrogen or deionized water for junctions with *G* > 1 nS (Figure S15). This medium dependence of the yield of stable junctions, and the transient nature of the instabilities seems to indicate that a significant cause for instabilities in our study might also be extrinsic due to inadvertent contamination of the system.

To investigate these processes, we suggest future studies where the gold surfaces are protected by a sacrificial layer until the microfluidic flow cell bonding is completed, more rigorous cleaning protocols for the gold surfaces before electromigration using mildly acidic or basic solutions, and the use of distilled solvents. These measures when combined with a larger number of devices will help determine whether the yield of stable devices at higher conductance (>1 nS), and of devices whose conductance stability persists over greater periods of time can be improved. Creating suspended electromigrated tunnel junctions with no supporting substrate might also help establish whether the dominant intrinsic source of conductance instability is the dielectric or the oxidized metal adhesion layer. These efforts would also help determine whether the dominant source of conductance instabilities is related to the material and geometry (and therefore intrinsic to the sensor) or to the conditions in which they were formed and studied. Such knowledge will enable further progress in developing robust on-chip tunnel junction sensors.

Another challenging aspect of performing molecule measurements in batches while measuring multiple devices in the same flow channel using a single channel electronic readout is how to carry out blank and sensing experiments consecutively on one device at a time, followed by a rinsing protocol to remove molecules before proceeding to the next device. One cannot be sure that the molecules of interest do not get permanently attached to the unmeasured devices even after the rinse, thereby interfering with a subsequent blank measurement. However, even this aspect could be resolved statistically, if the likelihood of the incidence instabilities in these junctions is known in a certain medium; a sudden increase in the frequency of devices showing high levels of fluttering following exposure to molecules even after rinsing might indicate the ineffectiveness of a rinsing protocol. Future studies will have to investigate these aspects closely.

## Conclusion

Using arrays of gold nanogap tunnel junctions manufactured by controlled electromigration and self-breaking, we showed that electromigration and self-breaking occur in mesitylene and deionized water in a manner largely indistinguishable from the same processes in air or nitrogen. To the best of our knowledge, this is the first time controlled electromigration and self-breaking have been demonstrated to occur under liquid immersion. A chromium adhesion layer used to achieve stable adhesion of gold to the SiO_2_ substrate did not obviously interfere with the electromigration process, and the morphology of devices formed compares well with the known results of pure gold electromigrated nanogap junctions. The yield of tunnel junctions was 44–48% when controlled electromigration and self-breaking were carried out in liquids, compared to 34–40% in nitrogen or 61–67% in air, indicating that there is some yield loss when performing electromigration under immersion in mesitylene or DIW compared to air but also a gain compared to nitrogen. Once formed, a significant proportion of tunnel junctions in all media studied with conductance less than 1 nS show stable conductance characteristics, whereas many junctions with conductance greater than 1 nS show moderate to high conductance fluctuation in pure media with no added molecules, especially in mesitylene. A closer study of this behavior in mesitylene reveals that several devices change from stable to unstable or vice versa when measured at different times, pointing to the possibility that a significant cause of conductance instability might be due to transient trapping of unknown molecules already in the system. However, intrinsic sources of conductance instabilities due to charge traps in the SiO_2_ layer or in the oxidized chromium adhesion layer cannot be excluded at this stage. Future studies using suspended electromigrated tunnel junctions or those focusing on a single liquid medium while testing the impact of different cleaning and conditioning protocols on the incidence of conductance instabilities will be required to clarify this issue before proceeding with label-free molecule sensing experiments using electromigrated gold tunnel junctions. In particular, understanding the contribution of the metal adhesion layer and substrate dielectric will be important for reliable on-chip nanogap sensor development. *I*–*V* sweeps in DIW show that tunnel junctions reconfigure to lower conductance abruptly at negative bias greater than 300 mV and that junctions which survive such treatment show a greater likelihood of stable conductance afterward. Such electrical stress could be a post-treatment step to achieve a higher yield of stable tunnel junctions in DIW for future studies. The high stability of active nanogap tunnel junctions of lower conductance already makes feedback-controlled electromigration and self-breaking a promising method to produce wider nanogaps for use with labeled sensing approaches such as recognition tunneling. In this regard many devices with too low conductance to be classified as tunnel junctions in this study would also be useful, with the possibility of increasing their relative proportion in the overall yield by running electromigration to higher target resistances (<1 *G*_0_) than used in this study or by cycling devices to high biases (>500 mV) after self-breaking is complete to trigger reconfiguration. Overall, the findings of this study open new avenues toward improved microfabricated on-chip nanogap-based single molecule sensors.

## Methods

### Wafer Preparation

A 100 mm diameter, 525 μm thick single-crystalline silicon wafer (100) with n-type doping and resistivity 2–5 mΩ·cm was used as a starting substrate. A 300 nm thick silicon oxide layer (SiO_2_) was thermally grown on the silicon wafer by wet oxidation. Then, a 2 nm thick chromium (Cr) adhesion layer and a 27 nm gold (Au) layer were evaporated using electron beam evaporation at a rate of 0.1 and 0.2 nm/s for Cr and Au, respectively, at a base pressure less than 5 × 10^–7^ Torr.

### Device Fabrication

For device fabrication, four main processes were carried out in sequence on the wafer scale: (1) patterning gold nanoconstrictions by dry etching, (2) patterning contacts by lift-off, (3) SiO_*x*_ deposition for dielectric passivation of devices, and (4) contact opening by patterned wet etching of the passivating layer. All photolithography was carried out with a projection stepper system (Nikon NSD TFHi12 I-line stepper) using a positive resist (SPR-700) after vapor phase HMDS coating of the wafer. Gold nanoconstrictions with a nominal width of 230 nm were fabricated by overexposing the resist mask using a dose of 180 mJ/cm^2^. The pattern was transferred to the deposited Cr/Au layer below by dry etching in an inductively coupled plasma (Oxford Instruments ICP 380, Ar 10 sccm, Cl_2_ 10 sccm, 5 mTorr, 100 W RF, 400 W ICP, 110 s etch duration). After resist strip using a combination of oxygen plasma, primarily to break down the resist sidewalls hardened by dry etching, and wet remover (mr-Rem 700, 5 min, last 1 min with ultrasonication), the wafer was cleaned in a spin rinse dryer and prepared for contact metallization. A stack of lift-off resists (LOR5A, Microchem) and SPR-700 were used at this step. It was found to be critical to perform HMDS coating to prevent delamination of the lift-off resist from the substrate below during the resist development step. A resist descum in an oxygen plasma (TePla Model 300 Plasma System, 200 sccm, 250 W, 5 min with a Faraday cage) to etch ∼50 nm of the resist layer was performed before depositing the metal contact pads (30 nm TiW adhesion layer and 170 nm Au) by sputtering (KDF 844NT). After lift-off was performed in acetone, to selectively dissolve the SPR-700 layer, the remaining resist was stripped off in remover (mr.Rem 700, 80 °C, 5 min) and cleaned in DIW and a spin rinse dryer. Immediately after cleaning in oxygen plasma (TePla Model 300 Plasma System, 200 sccm, 250 W, 2 min), SiO_*x*_ deposition by plasma-enhanced chemical vapor deposition (PECVD) was performed (Oxford Instruments Plasmalab 80 Plus, 250 °C, 95 s, 800 mTorr, 710 sccm 2% SiH_4_ in N_2_, 425 sccm N_2_O, 20 W) to achieve a thickness of ∼100 nm. The SiO_*x*_ passivation was patterned using photolithography, descum in oxygen plasma (to promote wetting of resist), and wet etching (130 s in 1:30 parts by volume mixture of 50% hydrofluoric acid and 40% ammonium fluoride). This produced 2 μm diameter circular openings centered at the nanoconstrictions, and large rectangular openings in the contact pads, to reveal the Au layer below. Finally, the front side of the wafer was protected by a layer of SPR-700, after which the thermal SiO_2_ layer on the backside of the wafer was fully removed by using etching in buffered hydrofluoric acid, followed by metallization of the backside of the wafer by using sputtered TiW/Au to facilitate subsequent grounding of the silicon layer of the wafer. Still protected by the same resist layer, the wafer was diced into individual chips (15 × 40 mm^2^ or 7.5 × 7.5 mm^2^). Then the individual chips were cleaned in remover, DIW rinse, isopropyl alcohol rinse, and dried with nitrogen. Finally, they were cleaned in oxygen plasma (TePla Model 300 Plasma System, 200 sccm, 250 W, 2 min) before the next step.

### Flow Cell Fabrication and Bonding

A 150 μm thick flow cell with through openings for electrical contact pads and 12,200 μm wide, 50 μm tall embedded fluidic channels with independent inlets and outlets was made of a thermosetting polymer, OSTEMER (OSTE 322, Mercene Labs AB). OSTE is resistant to most common solvents and showed little or no swelling during prolonged immersion (several days) in all the liquids used in the present study (deionized water, ethanol, mesitylene, and acetone). It is also impermeable to air when fully cured, leading to insignificant evaporation loss of liquid during long measurement periods.^66^ The complete details of OSTE flow cell fabrication by reaction injection molding and its bonding to the microfabricated chip with gold nanoconstrictions using aligned bonding under a stereo microscope are described elsewhere.^67^

### Sample Preparation for Measurements

The chip with the bonded OSTE flow cell was mounted onto the sample stage inside a Faraday cage and held in place using custom milled aluminum clamps and PDMS adapters with mating holes for the inlet and outlet fluidic ports in the OSTE flow cell. PTFE tubes (1.6 mm OD, 1 mm ID) were inserted into the PDMS adapter and held in place by an interference fit. While multiple channels could be perfused in parallel, we worked with a single channel at a time. The tubing, microfluidic channel, and the gold nanoconstrictions within were cleaned by perfusing acetone and then ethanol (200 μL/min, 15 min each), followed by drying by perfusing N_2_ gas (2 h) before perfusing the medium of the specific experiment. All liquid perfusion was carried out from outside the Faraday cage using a syringe pump, and microfluidic tubes crossed the cage through custom machined ports. As an unbroken fluid volume from outside the cage to the devices on chip introduced additional electronic noise from outside the cage into the electronic signal path, we perfused air through the tube until it crossed into the Faraday cage as a final step before measurement to eliminate this source of noise.^67^

### Electrical Measurements

Two probe connections to devices were made using a custom-made probe card with two miniature gold-plated spring-loaded pogo probes soldered to the tip at separation of 950 μm from each other, as described in detail elsewhere.^67^ Two versions of the probe card were made, which were identical in all ways except for the electrical terminals on one side customized to either accept (1) an SMB cable from the electrical feedthrough terminal on the Faraday cage wall to make shielded connection to a DC SMU (Keithley Sourcemeter 2450) outside the cage or (2) to directly mount a USB powered compact low current amplifier (LCA; Elements e1b) on the probe. Note that in the latter case the amplifier is inside the Faraday cage, and only a USB cable used for power delivery and data transfer crosses the Faraday cage. Using this arrangement, we minimized the parasitic capacitance to less than 1 pF while using the LCA.^67^ The pogo probes were aligned and positioned onto 100 μm wide open areas of the contact pads using a USB powered camera mounted on a parfocal lens for navigation. After probe positioning the motorized stage, which is also inside the Faraday cage, was powered down by software control to decrease the noise floor to the same level as when the stage was completed powered off by disconnecting it from the socket.^67^

Experiments unless otherwise noted were carried out in the following sequence: First electromigration was performed on a batch of devices, one device at a time, using a combination of control routines written in MATLAB as well as those running on the on-board processor of the DC SMU. After electromigration on all devices was complete, the probes were lifted by lowering the stage, and the devices were allowed to self-break typically for >12 h before subsequent measurements (the effect on yield of the self-breaking time is discussed in Supplementary Note 4). Next the DC SMU was disconnected from the cage and the LCA probe is connected, and subsequent measurements were performed using control routines running on MATLAB. The two most typical measurements using the LCA were (1) current–voltage (*I*–*V*) sweeps performed at 1.25 kHz sampling rate at a voltage scanning rate of 100 mV/s and (2) current traces acquired at a constant voltage bias, typically acquired at a sampling rate of 50–200 kHz. Several *I*–*V* sweeps were combined to compute *I*–*V* histograms of tunnel junction electrical characteristics. The USB camera was disconnected while performing measurements with the LCA. After initial alignment of the stage axis with the orientation of the arrays of devices on chip we could navigate blind.

Feedback-controlled electromigration was used to increase the resistance of a gold nanoconstriction gradually from a starting value of around 25 Ω to a final resistance of ∼1.3–2.6 kΩ (∼5–10 *G*_0_). This resistance increase was achieved over several voltage ramps, each starting at 0 mV and increasing in 10 mV steps at a rate of 50 mV/s until a predetermined resistance increase (5% per ramp until a 200 Ω, 10% thereafter) is measured, at which point the bias is immediately reset to 0 mV, after which the next ramp commences. The control routine we programmed to run on the onboard processor of the DC SMU could respond within 100 μs (compared to the 20 ms typical for USB communication to a PC), thereby largely eliminating the incidence of thermal runaway. After a certain resistance target was reached, a DC *I*–*V* sweep to ±200 mV voltage range was acquired before lifting the probes and moving to the next device. Based on this resistance measured immediately after electromigration, devices were classified as “On Target”, “Above Target” or “Runaways” as described earlier (Figure 2c).

Self-breaking data on some devices were collected using the DC SMU by applying a fixed voltage bias of 50 or 100 mV immediately after electromigration to a set resistance target was complete, recording the current trace either for 7200 s or until the conductance of the device was greater than 1 × 10^–3^*G*_0_.

### SEM Imaging after Electromigration under Liquid Immersion

The chip design for devices imaged in SEM after electromigration under liquid immersion (Figure 3e–p) was different than the standard design described earlier (Figure 1c) to achieve reversible flow cell bonding and removal. This also affects the heat sinking of the nanoconstriction, which in turn affects the cathodic offset of the nanogap formed after electromigration. These differences are described in the Supporting Information (Supplementary Notes 7 and 8 and Figures S8–S10).

### Parametrizing Stability Classification

*I*–*V* histograms constructed from 10 *I*–*V* sweeps of tunnel junctions recorded by using the LCA were used to assess the stability of the conductance characteristics of each junction. After manual expert labeling was performed to classify each device as stable or unstable, we identified four parameters computed from the *I*–*V* histograms, which would facilitate automation of this classification task. Mathematical notation is introduced next to explain the highest performing parameter (*F*_vc_) we identified to quantify conductance stability and to classify a junction as stable or unstable by identifying a threshold value. The relative performance and definition of all parameters are described in the Supporting Information (Supplementary Note 9 and Figure S15). We define *H* as the matrix containing bin counts from the *I*–*V* histogram. *H*_*i*|*v*_ signifies the bin count at current *i* and voltage *v* normalized by the total counts across all currents at voltage *v*. *v*_0_ represents the voltage of 0 V. Finally, *I* stands for the set of values of the current bins for a given device.

Then using the marginal probabilities, the mean and variance of the current for a given voltage can be expressed as
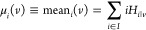
1
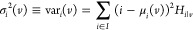
2The parameter which exhibited the highest accuracy and greatest class separation is
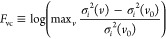
3

## Supporting Information Available

Table summarizing key statistics of all measurement batches, detailed description of feedback-controlled electromigration and self-breaking, description of characterization of feedback-controlled electromigration and self-breaking of Cr/Au devices in air, effect of self-breaking duration and medium of measurement on the conductance distribution of devices, SEM images of the progression of electromigration in air, description and illustration of devices used for SEM imaging after feedback-controlled electromigration and self-breaking under liquid immersion, effect of heat sinking on the cathodic offset of nanogaps, further details of parametric classification of stability of tunnel junctions from *I*–*V* characteristics, graphical summary of the experimental procedure and results from a measurement series in mesitylene (PDF)
