# Supplementary material for: Electromigrated Gold Nanogap Tunnel Junction Arrays: Fabrication and Electrical Behavior in Liquid and Gaseous Media
Source: ACS Appl Mater Interfaces. 2024 Jul 2;16(28):37131–46. doi: 10.1021/acsami.4c03282 (PMC11261569; doi:10.1021/acsami.4c03282)
Supplement: Supplementary file 1 — am4c03282_si_001.pdf [file am4c03282_si_001.pdf]

Supporting Information:

Electromigrated Gold Nanogap Tunnel Junction Arrays: Fabrication and Electrical Behavior in Liquid and Gaseous Media

Shyamprasad N. Raja[*,1,=], *Saumey Jain*[1,2,=], *Javier Kipen*[3], *Joakim Jaldén*[3], *Göran Stemme*[1], *Anna Herland*[2,4] *and Frank Niklaus*[+,1]

Email: [*]shnr@kth.se ; [+]frank@kth.se

[=] Equal contribution.

[1] Division of Micro and Nanosystems (MST), School of Electrical Engineering and Computer Science (EECS), KTH Royal Institute of Technology, SE-10044 Stockholm, Sweden.

[2] Division of Nanobiotechnology, SciLifeLab, Department of Protein Science, School of Engineering Sciences in Chemistry, Biotechnology and Health (CBH), KTH Royal Institute of Technology, SE-10044 Stockholm, Sweden.

[3] Division of Information Science and Engineering (ISE), School of Electrical Engineering and Computer Science (EECS), KTH Royal Institute of Technology, SE-10044 Stockholm, Sweden.

[4] AIMES-Center for the Advancement of Integrated Medical and Engineering Sciences, Department of Neuroscience, Karolinska Institute, SE-17177, Solna, Sweden

Table of Contents

TABLE S1: SUMMARY OF KEY STATISTICS OF ELECTROMIGRATED DEVICES.S3

SUPPLEMENTARY NOTE 1: DISCUSSION ABOUT THE IMPACT OF CHROMIUM ADHESION LAYER ON THE FEEDBACK-CONTROLLED ELECTROMIGRATION AND SELF-BREAKING PROCESS.S4

SUPPLEMENTARY NOTE 2: DESCRIPTION OF FEEDBACK-CONTROLLED ELECTROMIGRATION AND SELF-BREAKING.S5

SUPPLEMENTARY NOTE 3: CHARACTERIZATION OF THE ELECTROMIGRATION PROCESS FOR CR/AU DEVICES IN AIR.S6

SUPPLEMENTARY NOTE 4: EFFECT OF THE SELF-BREAKING DURATION ON THE CONDUCTANCE DISTRIBUTION OF DEVICES.S7

SUPPLEMENTARY NOTE 5: EFFECT OF CHANGING THE MEDIUM OF MEASUREMENT ON THE CONDUCTANCE DISTRIBUTION OF DEVICES.S11

SUPPLEMENTARY NOTE 6: TRACKING THE MORPHOLOGICAL PROGRESSION OF ELECTROMIGRATION IN AIR USING SCANNING ELECTRON MICROSCOPY.S14

SUPPLEMENTARY NOTE 7: DESCRIPTION OF CHIP ARCHITECTURE AND DEVICE DESIGN USED FOR SEM IMAGING AFTER ELECTROMIGRATION UNDER LIQUID IMMERSION.S17

SUPPLEMENTARY NOTE 8: EFFECT OF HEAT SINKING ON THE CATHODIC OFFSET OF THE NANOGAP AFTER ELECTROMIGRATIONS19

SUPPLEMENTARY NOTE 9: PARAMETRIC CLASSIFICATION OF THE CONDUCTANCE STABILITY OF TUNNEL JUNCTIONS FROM I-V CHARACTERISTICS.S27

SUPPLEMENTARY NOTE 10. CHARTS SUMMARIZING THE PROCEDURE AND KEY FINDINGS OF MEASUREMENT SERIES IN MESITYLENE.S30

Table S1: Summary of key statistics of electromigrated devices. Sample size, yield of total and stable tunnel junctions across all batches of measurements in various media presented in this work are summarized. All batch IDs shaded in blue are presented in Fig. 2 of the main text; all 8 batches feature in Fig. 5, 7 with the same batch IDs as shown here. The tunnel junctions featured in Fig. 8 originate from the Mes-B* batch.

Batch ID [a]	**Air**	**N2-A***	**N2-B***	**Mes-A**	**Mes-B***	**EtOH**	**DIW-A**	**DIW-A***
Medium	Air	Nitrogen		Mesitylene			Deionized water	
Number of electromigrated devices [b]	49	50	50	39	48	46	50	– [d]
Average self-breaking time [h]	~35	~18	~28	~33	~17	~26	~35	
Number of tunnel junctions	30	18	16 [c]	17	23	2	24	19
Number of <u>stable</u> tunnel junctions	7	9	7	1	7	0	3	12
Number of tunnel junction with $G > 1$ nS	22	13	12	16	12	1	13	12
Number of <u>stable</u> tunnel junction with $G > 1$ nS	1	5	4	0	1	0	0	7

[a] All batch IDs marked with * represent batches where I-V sweeps of tunnel junctions were performed to a maximum bias of ± 100 mV, to prevent their destabilization. In the unmarked batches I-V sweeps were performed to ± 500 mV. The batch ID Air used here is same as Air-A used in Fig. S1.

[b] This row represents the actual number of devices which where electromigrated out of a batch of 50 consecutive devices arrayed on chip; the deviation from 50 is because electromigration was only performed if a device was a low resistance (typically < 35 Ω) when measured after going in contact. If this was not the case, the electromigration was not carried out and the script automatically moved to the next device. Only in one batch (Mes-A) was the deviation from 50 significant– and even in this case it does not affect the overall outcome of the experiment, as shown in Fig. S1.

[c] One more tunnel junction in this batch could not be classified because it was measured using a current range which was too low, thereby saturation the amplified. This device was excluded from the count in this column.

[d] This column is a repeat measurement of device previously measured to ± 500 mV during I-V sweeps, and is not an independent set of devices.

Supplementary Note 1: Discussion about the impact of chromium adhesion layer on the feedback-controlled electromigration and self-breaking process.

Previous studies of electromigration, primarily in the field of molecular electronics, have typically focused on developing procedures to fabricate nanoconstrictions/nanowires with no adhesion layers over a 0.5–1 µm distance from where the electromigrated nanogap is expected to form[1,2]. This has been done so that metal-molecule contacts in these devices could be understood without the added complexity of metal intermixing in the electrode and to make comparisons with data from MCBJ or STM-BJ experiments using pure gold. Therefore, electromigration behavior of pure gold is well studied[3]. However, here we decided to use an adhesion layer because dielectric passivation was deemed important to achieve low noise in tunnel junction sensors operating in liquids[4], and because junctions had to operate in liquid for prolonged periods of time under immersion, both of which require good adhesion. The use of an adhesion layer raises three questions: (1) Would it adversely affect controlled electromigration? (2) Would it affect spontaneous self-breaking of electromigrated nanogap junctions to produce tunnel junctions? (3) Would it affect the stability of the tunnel junctions formed?

Past reports have shown that electromigration is possible in air/vacuum even while using titanium (Ti) or chromium (Cr) adhesion layers[5–7], and barring one exception[8], have treated the tunnel junctions so formed as no different from pure gold nanogap junctions. No studies have reported self-breaking data for such devices with adhesion layers. One study which compared electromigrated nanogap junctions made of pure gold with those made using a Ti adhesion layer, found that the gold devices were extremely unstable whereas the Ti devices were extremely stable under the application of voltage bias up to 1 V in air, attributing the stability to the tunneling gap being confined to a TiO_2 layer formed at the Ti/Au interface[8]. This in turn suggests that the morphology of a Ti/Au tunnel junction might not be suitable for dynamic molecule sensing, as the tunneling gap will not be a point contact exposed to the liquid, but rather a plane embedded in the metal layers forming the device. This picture is consistent with the finding of another study, which used transmission electron microscopy and X-ray photoelectron spectroscopy to investigate evaporated thin films, to show that Ti adhesion layers do not intermix with an overlayer of evaporated Au[9]. This same study also reported that a Cr adhesion layer intermixes with Au creating a 2–3 nm thick layer where a Cr-Au alloy is present. Therefore, when a Cr adhesion layer is used while forming a tunnel junction by electromigration it might have a significant effect on its behavior. Alloying is known to make a significant difference to the structural and electromigration properties of pure metals[3]. While specific information about the Cr/Au system is not available, alloying in general has been studied as a means to improve the electromigration resistance of metal interconnects in integrated circuits. Further, alloying has also been reported to affect the structural evolution of the electromigration process itself, with junctions forming by the creation and coalescence of voids, rather than the nucleation and ‘unzipping’ of a single gap running across the width of a junction in contrast to pure metal junctions[3,10].

We therefore first performed a baseline characterization of electromigration and self-breaking of our Cr/Au devices in air and determined that the behavior was indistinguishable from previous studies which used only gold without an adhesion layer. This is described in Supplementary Note 2.

Supplementary Note 2: Description of feedback-controlled electromigration and self-breaking.

Feedback controlled electromigration is the process by which the conductance of a nanoconstriction is decreased over several cycles of ramping the applied voltage bias such that a small decrease in conductance is achieved per cycle (Fig. 1d), with the aim of avoiding thermal runaway[11]. Electromigration is typically carried out to a level where the conductance of the junction reaches 5–10 times the quantum on conductance ($G_0 \sim 77.4$ μS) at which point the two sides of the junction are connected only by a nanowire a few atoms across. After this the conductance of the junction can spontaneously decrease in discrete steps (Fig. 1e) due to the migration of atoms forming the nanowire connection between the two sides of the junction, finally breaking to form a gap, which then makes up the tunnel junction[2,12]. Called self-breaking, this process has previously been shown to produce nanogap tunnel junctions free of issues such as coulomb blockade due to atomic islands of gold within the nanogap – which have been reported to occur when the formation of tunnel junctions is actively driven by an applied bias into the tunneling regime (i.e., conductance much less than G_0), rather than to its threshold resistance value (5–10 G_0).

Supplementary Note 3: Characterization of the electromigration process for Cr/Au devices in air.

To establish a baseline characterization of our devices, we first performed feedback-controlled electromigration experiments in air. We found that the electrical behavior of our Cr/Au devices under electromigration in air largely followed the behavior described for pure Au junctions[13]. Briefly, starting from a resistance of 25–30 Ω, the resistance of devices could be gradually increased to the desired resistance target of 1300 Ω or 2600 Ω over several voltage ramps, as shown in Fig. 1d. The first 2–4 ramps showed a decrease in total resistance, due to annealing of the polycrystalline metal layer around the nanoconstriction by joule heating from the passage of current. This is noteworthy considering that all devices have already undergone a short duration annealing at 250 ºC during the deposition of the dielectric passivation. In these initial ramps the stopping voltage of the individual ramps also increases peaking around 450–470 mV after which it monotonically decreases until reaching a cusp voltage around 200 mV as the total resistance reaches around 100 Ω. In most devices the stopping voltage then increases again, such that the current-voltage plot capturing the instant at which each electromigration ramp stops (IV_{Stop}), which we call the electromigration stopping trajectory, approximately follows a hyperbolic curve, in agreement with the critical power dissipation model proposed by Strachan et al.[11] (Fig. S6a). However, in several devices a single value of critical power cannot be identified for the entire electromigration range, but rather a decrease in critical power is observed as the resistance increases beyond the cusp voltage, also in agreement with previous reports in gold junctions[13]. The resistance during electromigration is very well controlled by the feedback process until the resistance reaches around 200 Ω, above which the resistance increases are less well controlled, often showing discrete jumps well in excess of the target set for a particular ramp. Despite this we were able to achieve a high yield of controllably electromigrating devices close to the set target resistance using a commercially available SMU with an on-board logic processor with a response time to the programmed feedback loop of ~ 100 μs. 46 out of 49 devices electromigrated in air reach a target resistance less than 10 kΩ, with the average final resistance normalized by the resistance target (1300 or 2600 Ω) being 1.06 ± 0.07.

Supplementary Note 4: Effect of the self-breaking duration on the conductance distribution of devices.

We did not systematically control for the precise self-breaking time in Figure 2. Empirically in our early testing in air on smaller batches we observed that the greatest change occured during the first few hours after electromigration and that after 12 h the overall conductance distribution does not change remarkably. In Figure S1 we present a full listing of the outcome of the electromigration and self-breaking process in different batches of devices in various media also showing the average self-breaking time in each case. We observe that the yield of tunneling devices was very similar across multiple batches in the same medium and is relatively insensitive to the self-breaking time > 12 h. While making comparisons it is useful to consider that for a batch of 50 devices a change of one device in a certain category changes the proportion by 2 %.

Regarding the early stages of the self-breaking process, we present a more detailed breakdown of the set of 50 devices measured after ~ 9 h (N2-A) and ~ 28 h (N2-A*) of self-breaking in Figure. S2. The most significant change occured to the high G and low G devices (Fig. S2a), while the overall spread of conductance of tunneling devices remained similar (Fig. S2b). During these measurements, of the 43 devices which were high G or tunneling after ~ 9 h, 38 devices showed a decrease in conductance when measured at ~ 28 h, of which 21 devices showed a more than 10x decrease (Fig. S2c). Therefore, there is ~ 49 % chance of a 10x decrease in conductance for a device in nitrogen measured ~ 19 h apart, with the first measurement having been done after a < 12 h self-breaking period.

Such large changes in conductance became less prevalent if self-breaking was allowed to occur for a period of > 12 h. For instance, if we consider two different sets of measurements of 48 devices in mesitylene ~ 17 h and ~ 39 h after self-breaking (Fig. S3a, b), we observed that 30 out of 48 devices which were either high G or tunneling after the first measurement, 19 devices showed a decrease in conductance, of which only 7 devices showed a greater than 10x decrease in conductance (Fig. S3c). Thus, there is only a 23 % chance of 10x decrease in conductance of a device between these two measurements in mesitylene performed ~ 22 h apart.

In summary, when a self-breaking time longer than 9 h was used, the number of devices classified as tunneling in subsequent measurements changed by less than 8 % in all the media studied and the overall spread of conductance of tunneling devices was similar. While the conductance of an individual device continued to change, typically decrease, most of this change occured in the first 12–24 h of self-breaking.

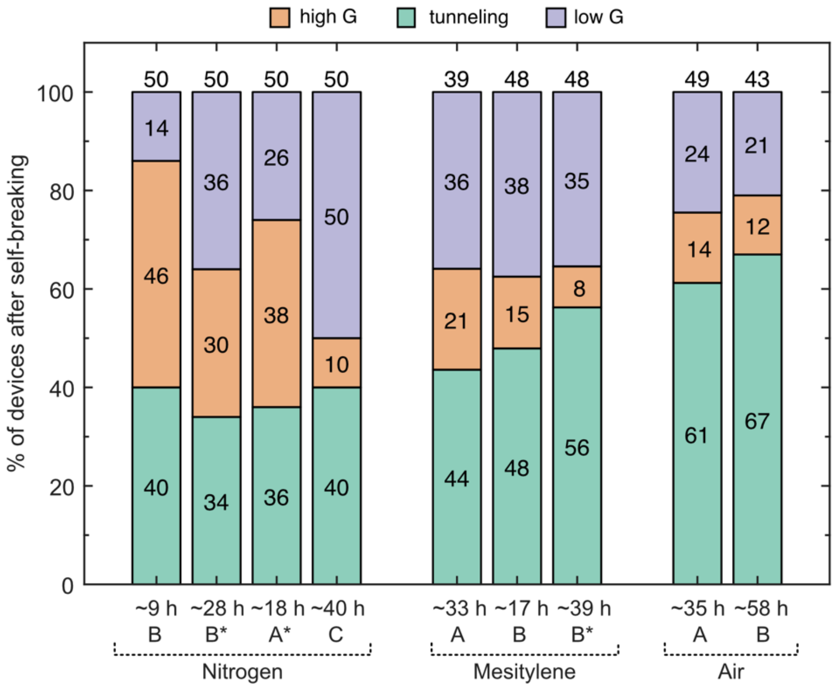

Figure S1. Repeatability of electromigration and self-breaking in nitrogen, mesitylene and air. Bar graphs show the outcome of the self-breaking process after electromigration for different self-breaking durations in nitrogen mesitylene and air. Devices are grouped into three categories based on their conductance as described in the main text and in Fig. 2. The numbers inside each bar graph segment show the percentage of devices in each category. The number at the top of each bar graph is the total sample size of devices considering all three categories. The yield of tunneling devices is very similar across multiple batches in the same medium and is relatively insensitive to the self-breaking time > 12 h. Note that Nitrogen-A*, Mesitylene-A and Air-A have already been presented in the main text and Fig. 2b.

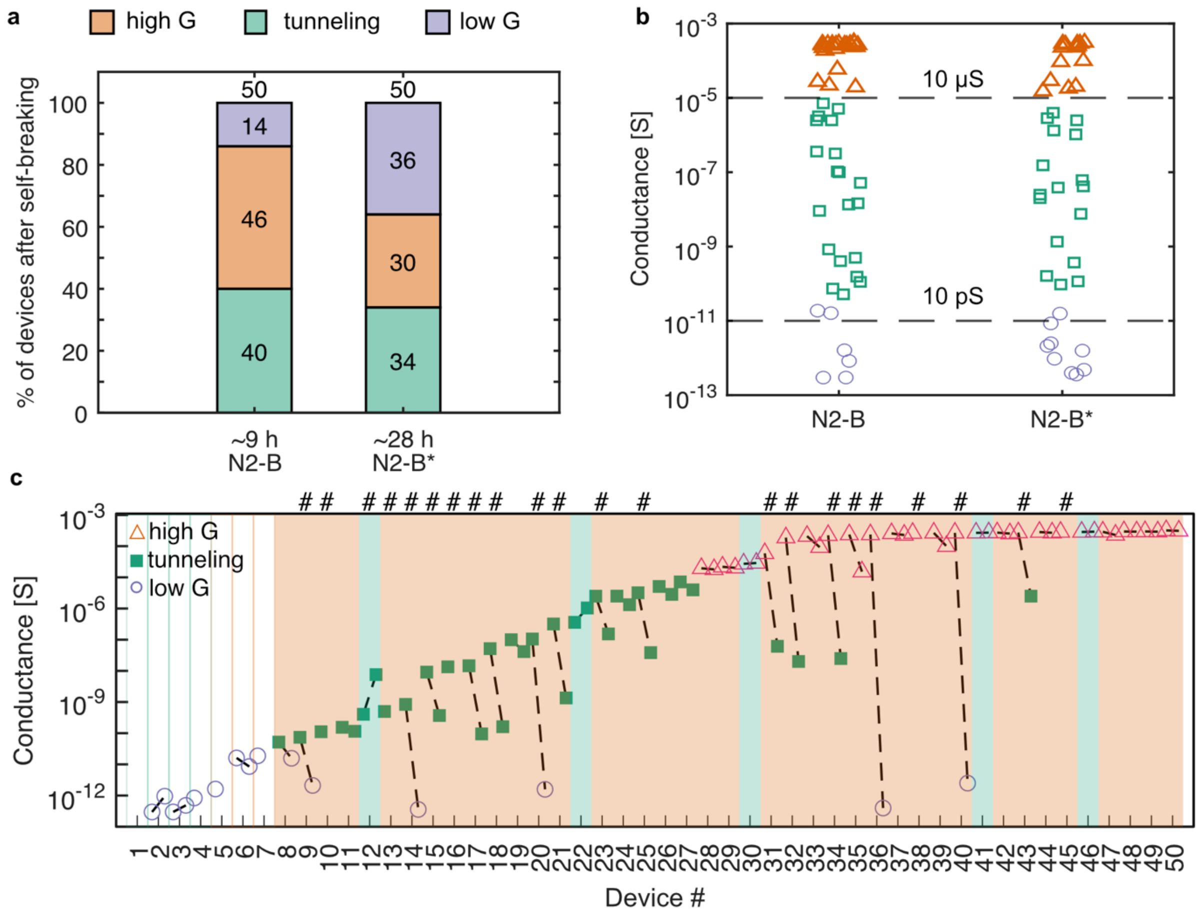

Figure S2. Tracking the early stages of self-breaking in nitrogen. (a) Bar graphs showing the outcome of the self-breaking process after electromigration in one batch of devices in nitrogen measured after self-breaking periods of ~ 9 h and ~ 28 h. The devices are grouped into three categories based on their conductance as described in the main text and in Fig. 2. The numbers inside each bar graph segment show the percentage of devices in each category. The number at the top of each bar graph is the total sample size of devices considering all three categories. (b) Conductance of all devices after self-breaking. The marker color indicates the conductance category (high G, tuneling or low G). (c) Conductance of all 50 devices measured at two time points, sorted from left to right in ascending order of conductance in the first measurement of the series. Different measurements of a specific device are arranged consecutively from left to right and connected by a dashed black line. The marker type indicates the conductance category of a device (low G, tunneling, or high G). The column color of a specific device indicated whether the conductance increased (green) or decreased (orange). All devices showing a > 10x conductance change during the series are marked with # at the top of the column. We observe that of the 43 devices initially classified as tunneling or high G, 38 devices showed a decrease in conductance, with 21 devices showing a decrease of more than 10x of the initial conductance.

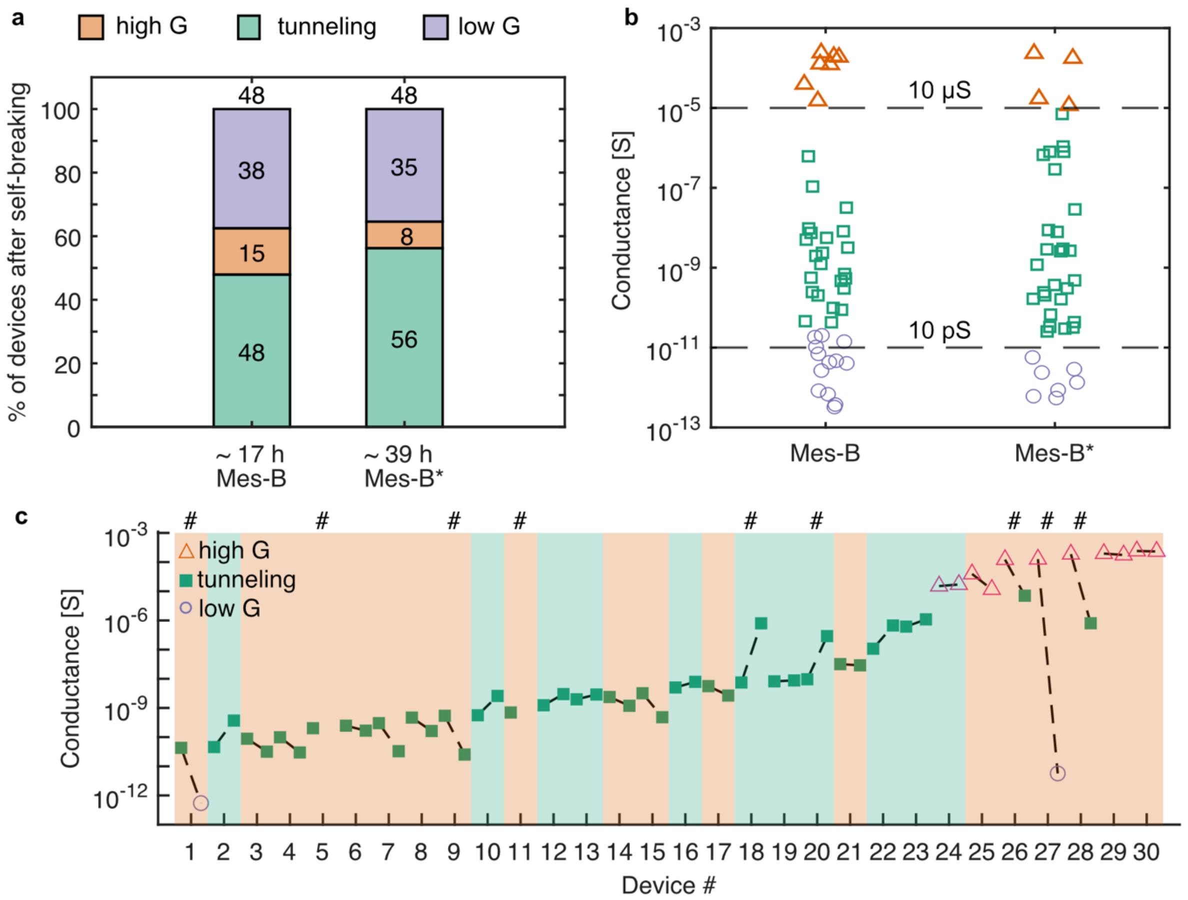

Figure S3. Tracking conductance changes in mesitylene after a long self-breaking period. (a) The conductance classification and (b) conductance distribution of devices electromigrated in mesitylene and allowed to self-break for ~ 17 h before the first measurement; ~ 22 h elapse between the two measurement sets. (c) Conductance changes were much less pronounced since self-breaking was allowed to occur for > 12 h before the first measurement. Of the 30 devices which were not low G initially, 19 devices showed an increase, and 11 devices showed a decrease in conductance. Different measurements of a specific device are arranged consecutively from left to right and connected by a dashed black line. The marker type indicates the conductance category of a device (low G, tunneling, or high G). The column color indicates whether the conductance increased (green) or decreased (orange). Devices showing a > 10x conductance change are marked with # at the top of the column.

Supplementary Note 5: Effect of changing the medium of measurement on the conductance distribution of devices.

In Fig. 2a,b we show the yield and subsequent classification of devices into three categories based on their conductance by performing current-voltage (I-V) sweeps after a certain self-breaking period (> 24 h) while keeping the immersion medium unchanged. This is to estimate the representative yield of the entire process of electromigration and self-breaking performed in the same medium. The conductance of an individual device is expected to vary depending on the medium of measurement. For example, in the case of gold break junctions the geometry of the junction itself has been found to heavily influence the actual measured conductance variation[14]. In previous studies of fixed-gap tunnel junctions where the same device was characterized in various media the conductance changes of a certain device typically varied by less than an order of magnitude between measurements[15], or when larger changes were observed these changes did not correlate with the expected change based on the relative barrier heights of the media studied[16]. Based on our findings, changes greater than an order of magnitude can typically be attributed to uncontrollable electrode reconfigurations probably occurring during the change of medium rather than a purely electrical response due to a change in barrier height. To our knowledge, no one has presented data to show reversibility of such conductance changes using fixed gap tunnel junctions of atomic scale contacts when returning to the original medium (in contrast to those made by planar electrodes at nanometric separation[17]). This is likely due to the difficulty of removing adsorbates from the electrode tips, which is the reason tunnel junctions made under ambient conditions typically show barrier heights less than 2 eV even when measured in vacuum, well below the work function of gold (5 eV)[18].

In Figure S4 we show measurements of 48 devices electromigrated in mesitylene, measured after ~ 39 h of self-breaking and then measured again in nitrogen ~ 3 h later. The channel was cleaned by perfusing ethanol and then dried under nitrogen flow for 2 h before the measurements in nitrogen were performed. We saw a 10 % change in the proportion of tunneling devices (Fig. S4a). The overall conductance distribution was very similar in both media (Fig. S4b). A more detailed analysis of the conductances of 31 devices which were high G or tunneling (i.e., low G devices are not shown) reveals that 5 devices show a > 10x change in conductance (# marked columns in Fig. S4c). Decrease in conductance is observed in 18 devices (orange columns) and increases in 13 devices (green columns).

The exact distribution of conductance of tunnel junctions (which span 6 orders of magnitude in conductance) is not expected to be the same from batch to batch even in the same medium, although they are very similar, as devices spanning the entire range of conductance have been observed in all media except ethanol. Achieving a tighter distribution of conductance of tunnel junctions remains an outstanding challenge for the fabrication of fixed-gap tunnel junction sensors.

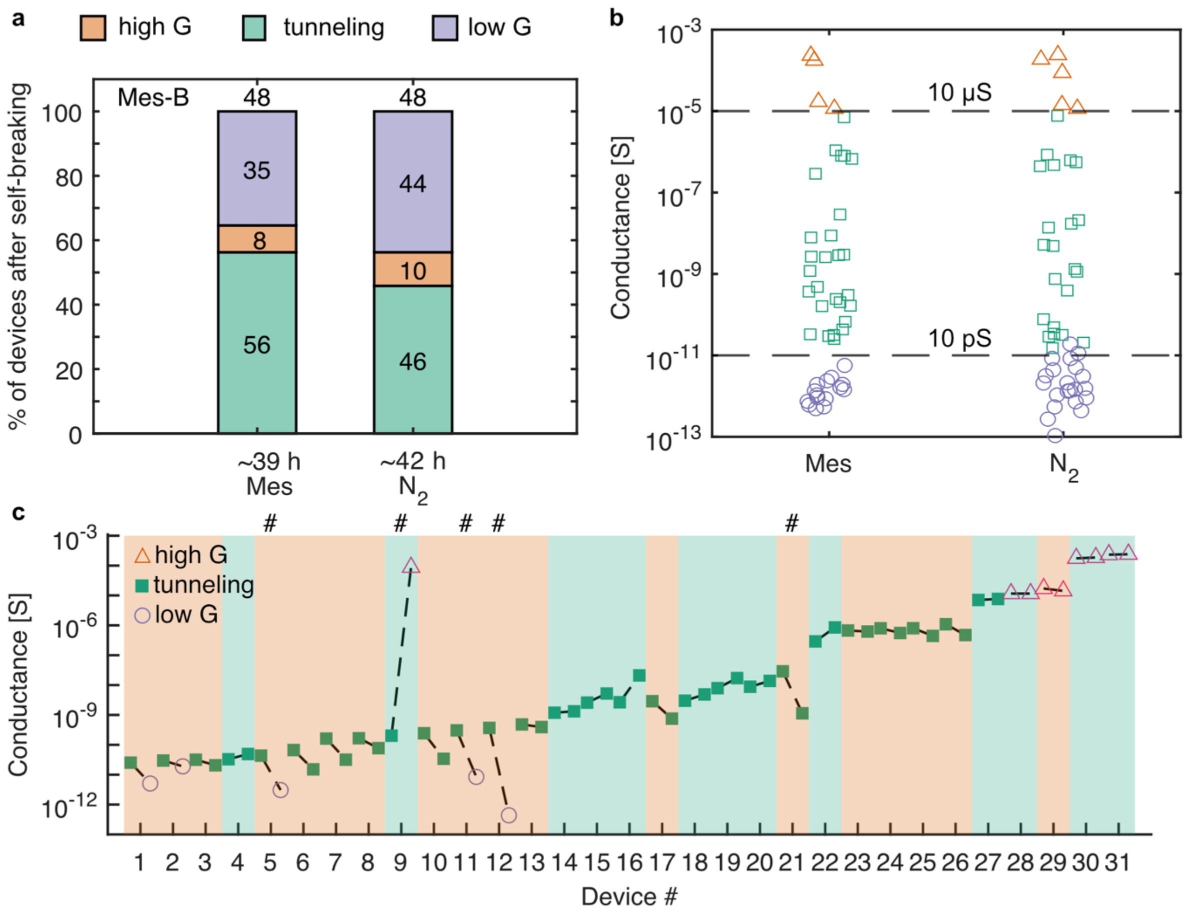

Figure S4. Influence of the measurement medium on device conductance when changing from mesitylene to nitrogen. (a) Conductance classification and (b) scatter plot of conductance showing the conductance distribution of the same set of devices measured in mesitylene and then in N_2 about 3 h later. The devices are grouped into three categories (high G, tunneling and low G) based on their conductance as described in the main text and in Fig. 2. The numbers inside each bar graph segment show the percentage of devices in each category. The number at the top of each bar graph is the total sample size of devices considering all three categories. (c) Conductance of 31 devices measured in two batches, sorted from left to right in ascending order of conductance in the first measurement of the series. The 17 devices which were low G in the first measurement are not shown. Different measurements of a specific device are arranged consecutively from left to right and connected by a dashed black line. The marker type indicates the conductance category of a device (low G, tunneling, or high G). The column color indicates whether the conductance increased (green) or decreased (orange). Devices showing a > 10x conductance change are marked with # at the top of the column.

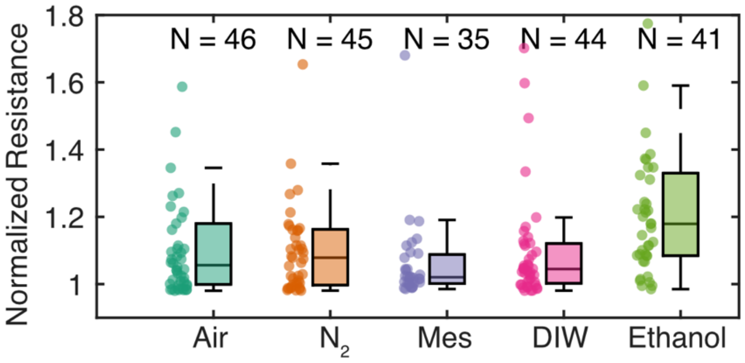

Figure S5. Box plots of device resistances after controlled electromigration in various media for devices 'on target' after electromigration. The data points used to generate each box plot are shown adjacent to the respective box, and the total number of 'on target' points (N) is also indicated for each case. This plot supplements data presented in Fig. 2c in the main text.

Supplementary Note 6: Tracking the morphological progression of electromigration in air using scanning electron microscopy.

To image the structural evolution in air of the nanoconstrictions by scanning electron microscopy (SEM) a series of nominally identical devices were electromigrated to various resistance targets. During the initial electromigration ramps, when the total resistance decreased slightly from the starting value, no significant change in the geometry of the device was observed (Fig. S6b). However, the nanoscale morphology of the polycrystalline surface of the evaporated metal layer was slightly altered, appearing smoother compared to the adjacent regions, consistent with local annealing and grain growth, which would also explain the initial decrease in resistance[3]. Already at the stage when the total resistance has increased by only 5 Ω from the starting value, we observed a clear slit opening across the width of the device, 200-250 nm away from the center of the nanoconstriction offset towards the cathode side (Fig. S6c). In all cases we observed that the slit had opened from both longitudinal edges of the device with the remaining connection somewhere away from the edges. After a resistance increase of only 10–15 Ω from the initial value, the two sides are only connected by one or at most two nanowires no more than ~ 10–20 nm across, indicating that the bulk of the migration of material was already completed in the first ten ramps of the process (Fig. S6d, e). By the time the resistance has increased to 100–150 Ω the ohmic connection between the two sides was no longer clearly visible (Fig. S6h). And devices with resistances higher than this could not be easily differentiated based on their resistances by SEM imaging. In 4 of 32 imaged devices a single isolated void offset towards the cathode side with respect to the primary slit was observed (Fig. S6i, left edge of image). In 5 of 32 devices a partially opened second slit towards the anode side was observed (Fig. S6f). In nearly all devices the expected location of the nanogap which creates the tunnel junction could be identified. Overall, the behavior of our Cr/Au junctions during electromigration in air is indistinguishable from previous reports of pure Au junctions[3,13], therefore any effect of alloying does not make a difference until this point of the process.

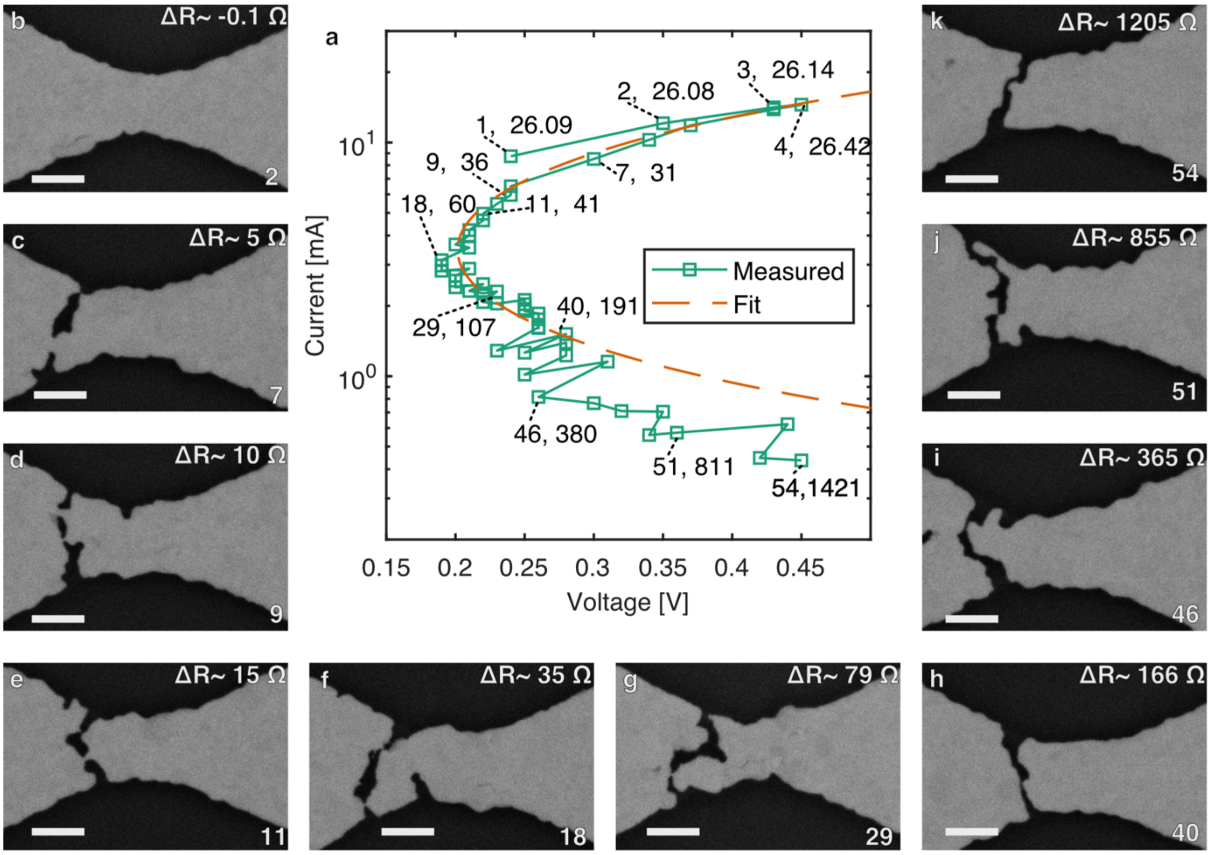

Figure S6. Progression of controlled electromigration in air. (a) The current and voltage values at the end of each electromigration ramp, collectively referred to as IV_{Stop}, for a single device migrated to 1421 Ω from a starting resistance of 26.1 Ω over 54 ramps is shown. Selected points are labeled with two numbers indicating the ramp number and the resistance at the end of the ramp. The resistance after the first two ramps is less than the starting value. IV_{Stop} increases until the fourth ramp after which electromigration becomes the dominant phenomenon and the curve follows the hyperbolic trajectory defined by the constant power dissipation model for a critical power of 350 μW (red dashed line). Notice that the measured values deviate significantly from the model as the resistance increases above ~ 200 Ω for this device. (b)-(k) SEM images of devices electromigrated to various resistances in air. Each image is a different device. The actual resistance change (ΔR) from the starting resistance of each device is shown. The ramp number on the IV_{Stop} curve shown in (a) to which the ΔR matches best is also mentioned at the bottom right of each panel. Scale bars for parts b–k is 200 nm. All SEM images were collected using an ESB detector which is sensitive to backscattered electrons.

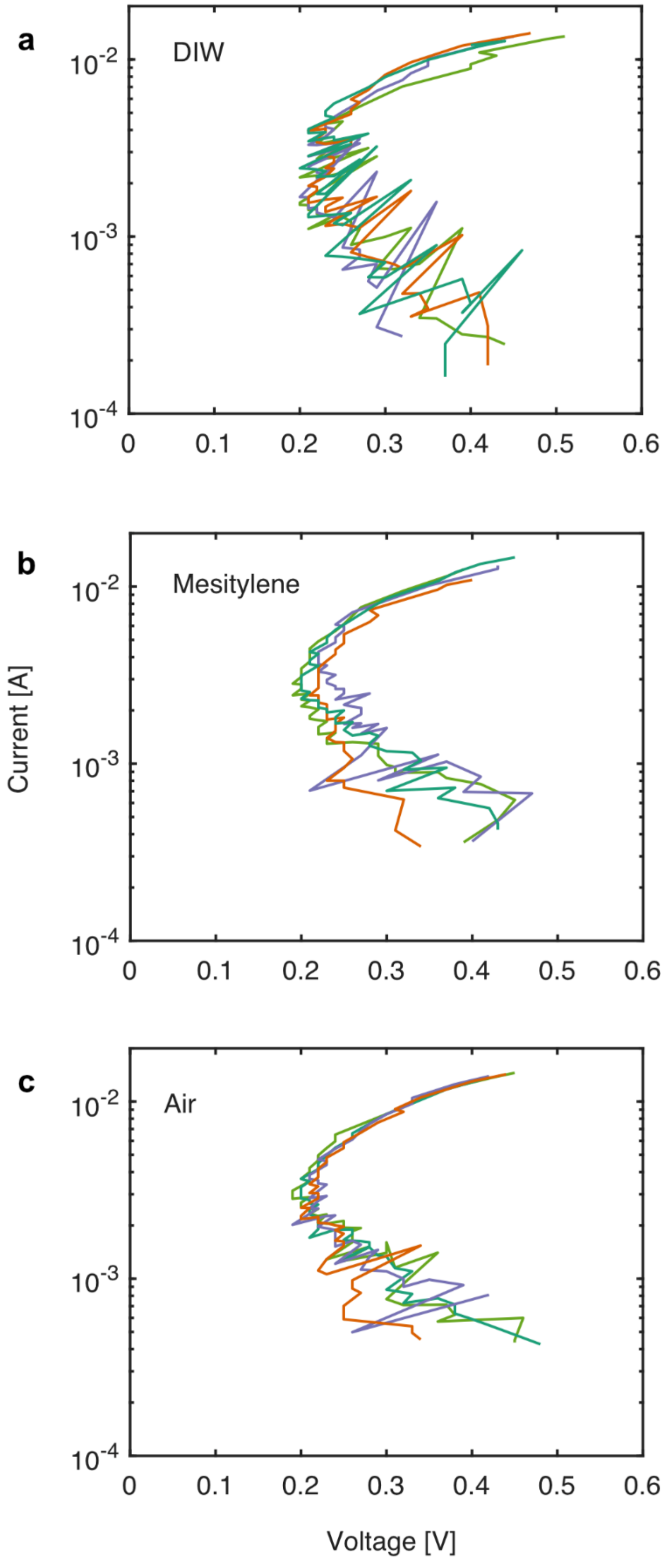

Figure S7. Electromigration stopping trajectories (IV_{Stop}) for four devices in DIW, mesitylene and air. (a) Switching between an inner and outer trajectory for these devices electromigrated in DIW can be seen, producing the 2D histogram observed in Fig. 2g. Such switching was not typically observed in (b) mesitylene or (c) air, shown here for comparison.

Supplementary Note 7: Description of chip architecture and device design used for SEM imaging after electromigration under liquid immersion.

A different chip architecture and device design (Fig. S8) was used to study the effect of electromigration under liquid immersion using SEM imaging. In this case we wanted to use a simple PDMS flow cell which could be temporarily bonded to the chip for the electromigration experiments, and which could be removed afterwards for SEM imaging. To achieve this the nanoconstrictions were arrayed in a circle around a common shared electrode (which served as the cathode in the experiment), whereas the devices used for electrical investigations alone had no common electrodes (i.e., each device had two independent electrical terminals). Also, the thicker contact lines extended up to 2.5 μm from the center of the nanoconstrictions, whereas they stopped 15 μm away from the center in the standard design. This has a noticeable effect on the heat sinking of the nanoconstriction during electromigration which is discussed in Supplementary Note 8. The circular layout of these devices with a common electrode meant the chips could be bonded to a flow cell by hand using a simple stereo microscope, but it was not suitable for performing long duration measurements due to evaporative loss of liquid through PDMS. Moreover, the long lead lines and large wetted area resulted in at least ten times higher electronic noise and was therefore not preferred for high bandwidth measurements either.

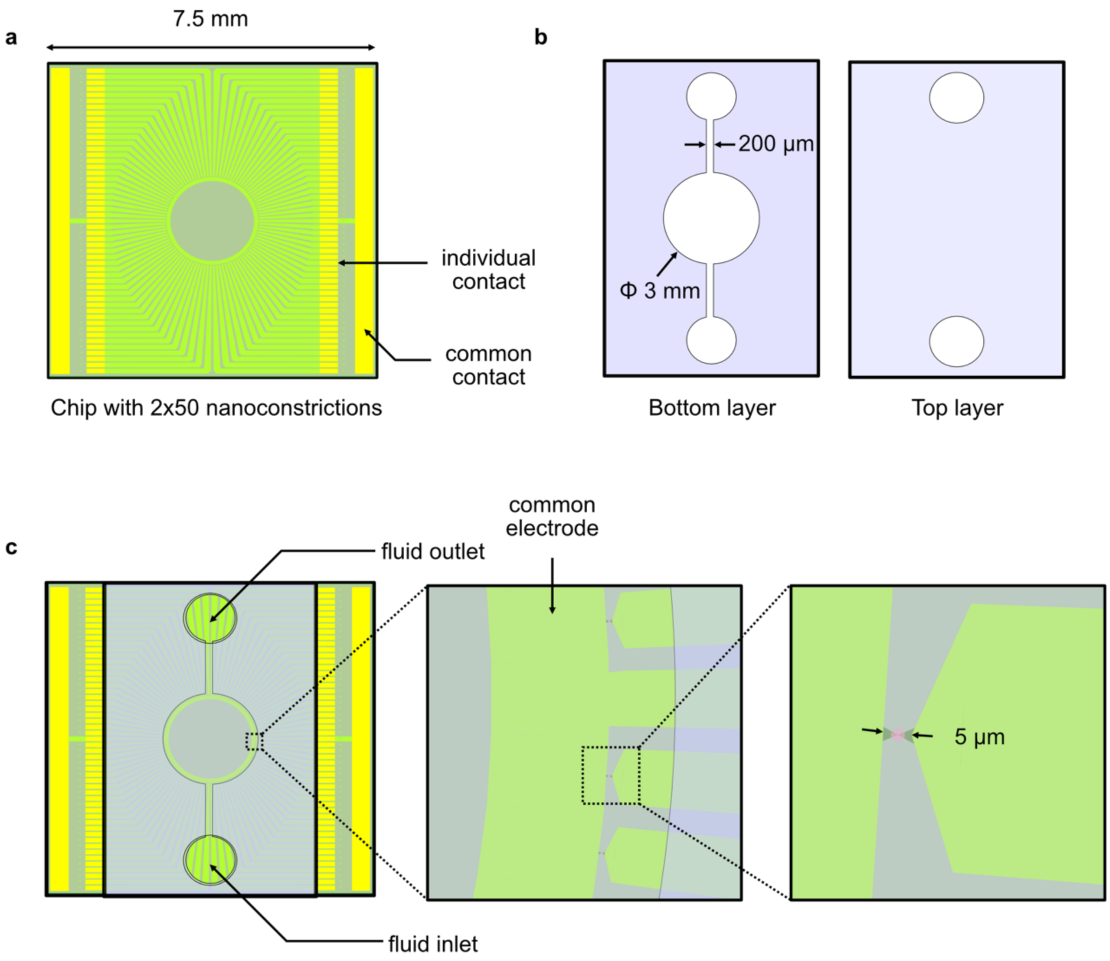

Figure S8. Overview of chip architecture and device design used for SEM imaging after electromigration under liquid immersion. This chip architecture allowed reversible PDMS flow cell bonding to carry out SEM imaging after electromigration to different resistance targets in liquids. (a) 7.5 x 7.5 mm chip containing an array of 100 gold nanoconstriction devices arrayed around a circle 2 mm in diameter. All devices share a common ring electrode. 50 devices each can be contacted using individual contact pads arrayed along and the left and right sides of the chip. (b) A double layer PDMS flow cell was bonded to the chip such that in the final assembly (c) only a 3 mm diameter circular area of the chip was wet. The magnified views show how in this design the thick gold electrodes extend to within 2.5 µm of the center of the nanoconstriction.

Supplementary Note 8: Effect of heat sinking on the cathodic offset of the nanogap after electromigration

While wind force is the dominant force in electromigration of gold causing the cathodic offset of the nanogap, the exact location of the nanogap is also dependent on other factors[3]. Using an FE model to compute the temperature and current-density distribution of a nanoconstriction undergoing electromigration, Kießig et al. showed that the total thermal resistance of the device, which includes the thermal resistance due to the thicker metal leads in the vicinity of the nanoconstriction as well as due to the substrate through the insulating dielectric below, plays a significant role in determining the final location of the nanogap formed due to electromigration[19]. Specifically, they showed that when the thermal resistance is very low, it is possible to determine the location of nanogap formation using nanoconstrictions in the metal more precisely than when the thermal resistance is high.

In our experiments we observed the effect of heat sinking on the cathodic offset of the nanogap created by electromigration by varying the thermal resistance of the nanoconstriction in two ways: (1) changing the SiO_2 thickness (*t*) of the substrate (100 nm and 300 nm, respectively); (2) changing the metal layout to change the distance (*d*) between the thick metal lead lines and the center of the nanoconstriction (2.5 µm and 15 µm, respectively). Decreasing *d* or *t* decreases the thermal resistance and make heat-sinking more effective. The three types of samples studied by varying these two parameters is schematically shown in Fig. S9a. The way the cathodic offset of nanogaps after electromigration was measured from SEM images is shown in Fig. S9b, and the offset values using the different geometries in various media is shown in Fig. S9c.

When the SiO_x thickness was decreased from 300 nm to 100 nm (for $d = 15$ µm in both cases) we observed a significant decrease in the cathodic offset for samples electromigrated in air. Similarly, the cathodic offset of samples with $d = 2.5$ µm (in DIW or Mesitylene) was significantly lower than that where $d = 15$ µm (in air) ($t = 300$ nm in both cases), indicating that the cathodic offset was decreased by bringing the metal lead lines closer to the nanoconstriction. When comparing samples with identical geometries electromigrated in DIW and mesityele, we observed that while both show a similar spread of cathodic offset, there is a significant cluster of samples in mesitylene which produced a smaller cathodic offset than in DIW. This indicates the possibility of additional heat-sinking due to mesitylene, by conductive and convective heat transport, presumably enabled by its higher boiling point compared to DIW– meaning that it was less likely to locally vaporize, which in turn would drastically increase the thermal resistance due to this mode of heat transfer.

In addition to the cathodic offset of the nanogap, the effect of heat sinking can be observed in the region where significant recrystallization of the metal was observed in SEM images (Fig. S10). In samples with thinner SiO_x or where the lead lines were close to the nanoconstriction, very little recrystallization was observed and the granularity of gold is evident (Fig. S10e, g; Fig. 4e–g); whereas in the configuration used for all our electrical characterization studies significant recrystallization immediately adjacent to the nanogap was evident (Fig. S10d).

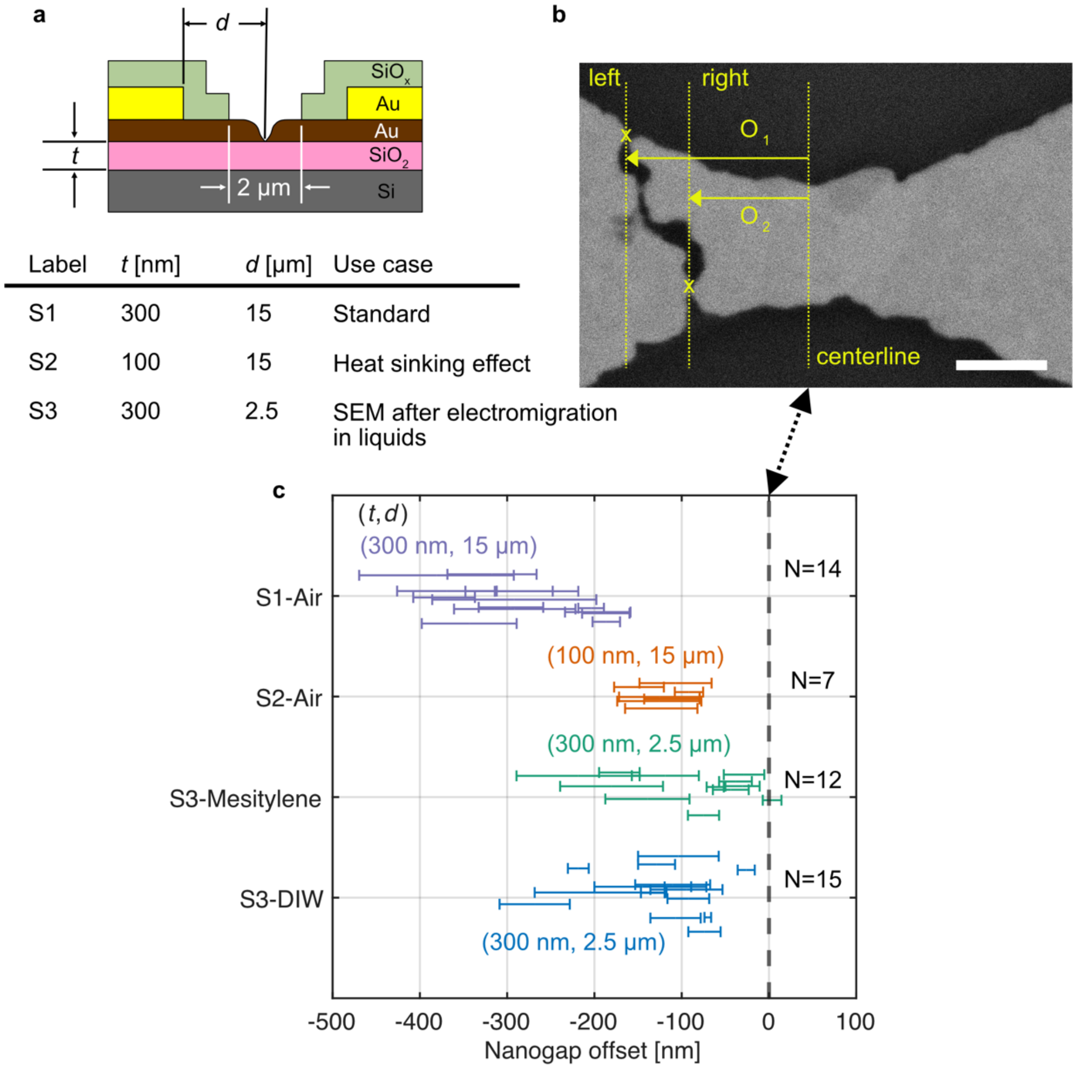

Label	t [nm]	d [µm]	Use case
S1	300	15	Standard
S2	100	15	Heat sinking effect
S3	300	2.5	SEM after electromigration in liquids

Figure S9. The influence of heat sinking and the immersion medium on the magnitude of cathodic offset of the nanogap formed by electrmigration. (a) Cross-section schematic and table outling the three different sample types evaluated (S1, S2 and S3) by varying two design parameters: (1) the SiO_x thickness(*t*) or (2) the distance between the center of the nanoconstriction and the thick gold contact lines (*d*). Decreasing *t* or *d* would make heat sinking more effective. (b) SEM image showing how the offset of a nanogap produced by electromigration was characterised by two different offset measures (O_1, O_2) representing the leftmost and rightmost extents of the left edge of the nanogap. X marks the point on the lines labeled left and right which is the precise location demarcating these extents in this sample. Scale bar is 200 nm. (c) Nanogap offset measurements grouped by the sample type and medium of measurement. Each sample is represented as a horizontal errorbar whose left and right extents represent the O_1 and O_2 values respectively. A narrow (O1,O2) range for a particular sample indicates the formation of a near vertical nanogap, whereas a wider range indicates a more slanting nanogap.

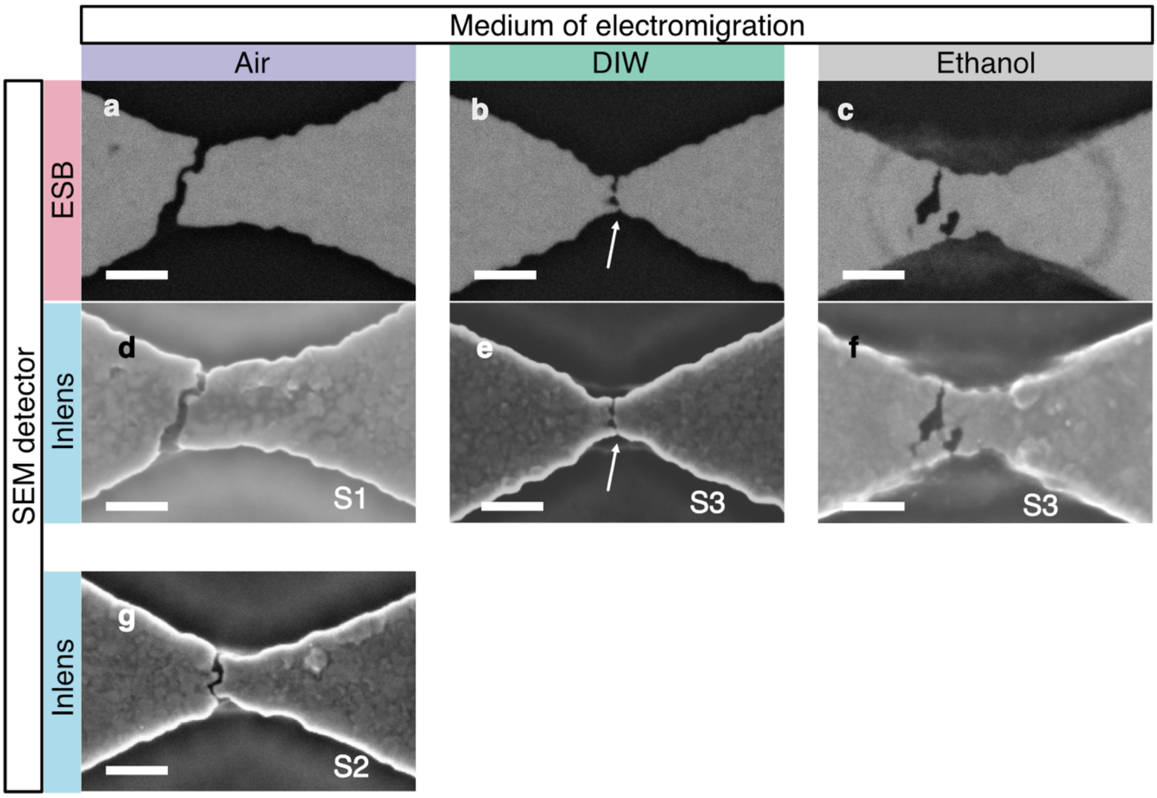

Figure S10. SEM images from various sample types electromigrated in air, DIW and ethanol. Sample labels S1–S3 are same as in Fig. S9. (a)-(c) Images collected using the ESB detector which is sensitive to back-scattered electrons were better at showing the presence or absence of gold. This is because the high atomic mass of gold makes it more likely to backscatter incoming electrons. (d)-(f) Corresponding images of the same area in (a)-(c) collected using the Inlens detector which is sensitive to secondary electrons and is better to resolve surface details such as the granularity of the gold layer. However due to differences in sample clamping on the SEM stage and sample charging during SEM imaging as well as carbon contamination it was challenging to maintain uniform contrast across samples using the Inlens detector. For instance, the arrows in (b) and (d) show a location near the edge of the nanoconstriction where a gold nanowire is clearly seen in the ESB image whereas edge contrast obscures this information in the Inlens image. Therefore we preferred using the ESB detector to track the progress of electromigration. (g) Inlens image of an electromigrated sample of type S2, showing that the recrystallization of gold in the vicinity of the electromigrated nanogap is much less pronounced as compared to that in an S1 type sample (see panel d) due to the more efficient heat sinking of a thinner SiO_x substrate layer. Similarly, less pronounced recrystallization in S3 type samples (see panel e) due to the heat sinking effect of closer lead lines is observed. All scale bars are 200 nm.

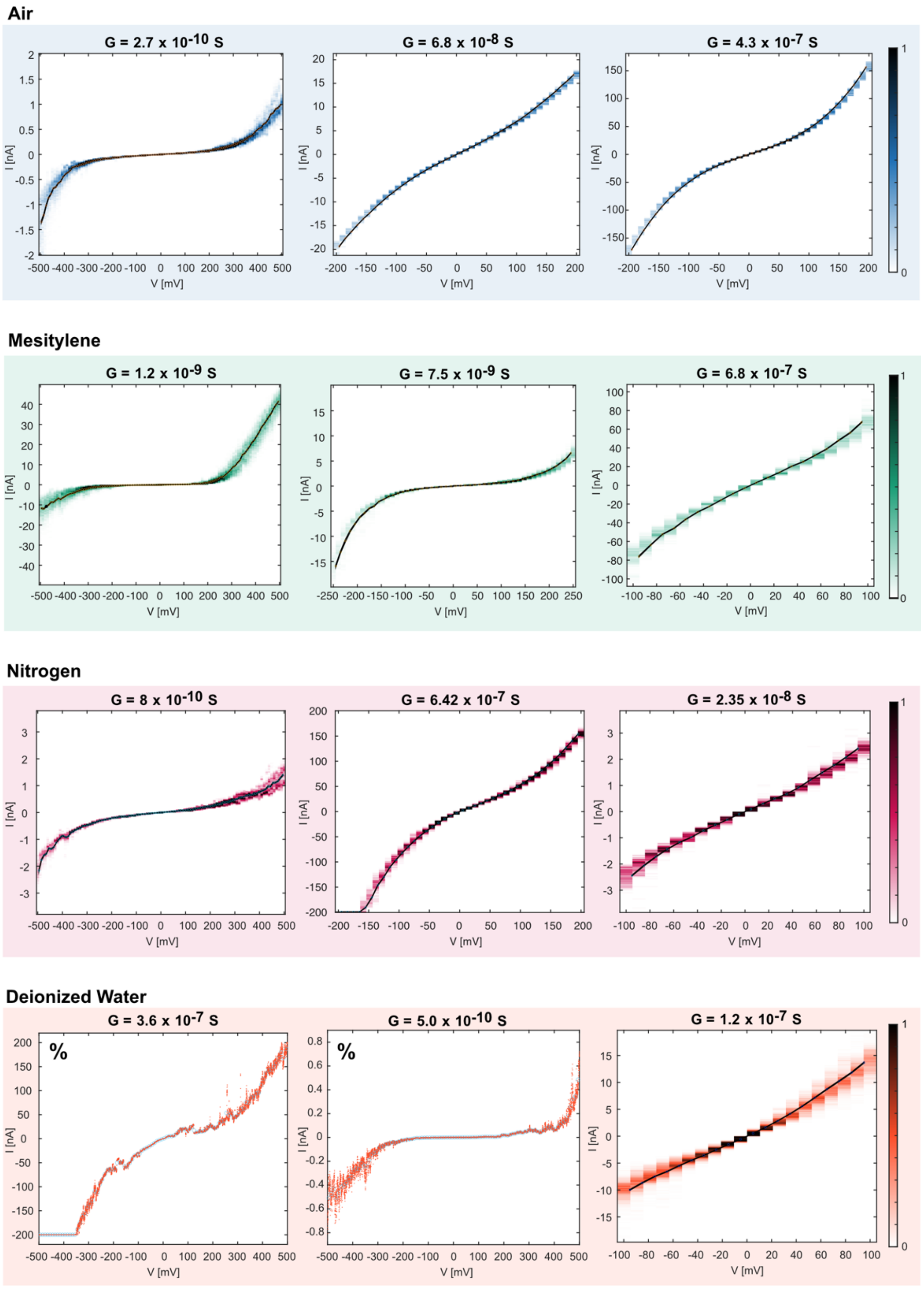

Figure S11. S-shaped I-V curves of tunnel junctions observed in air, mesitylene, nitrogen and deionized water (DIW). I-V histograms of 10 I-V sweeps overlaid with a 10 mV resolution binned average I-V sweep are shown. In the two panels marked with % due to device reconfiguration the raw data of a single sweep collected at a sampling rate of 1.25 kHz at a sampling rate of 100 mV/s overlaid with the binned average I-V sweep are shown.

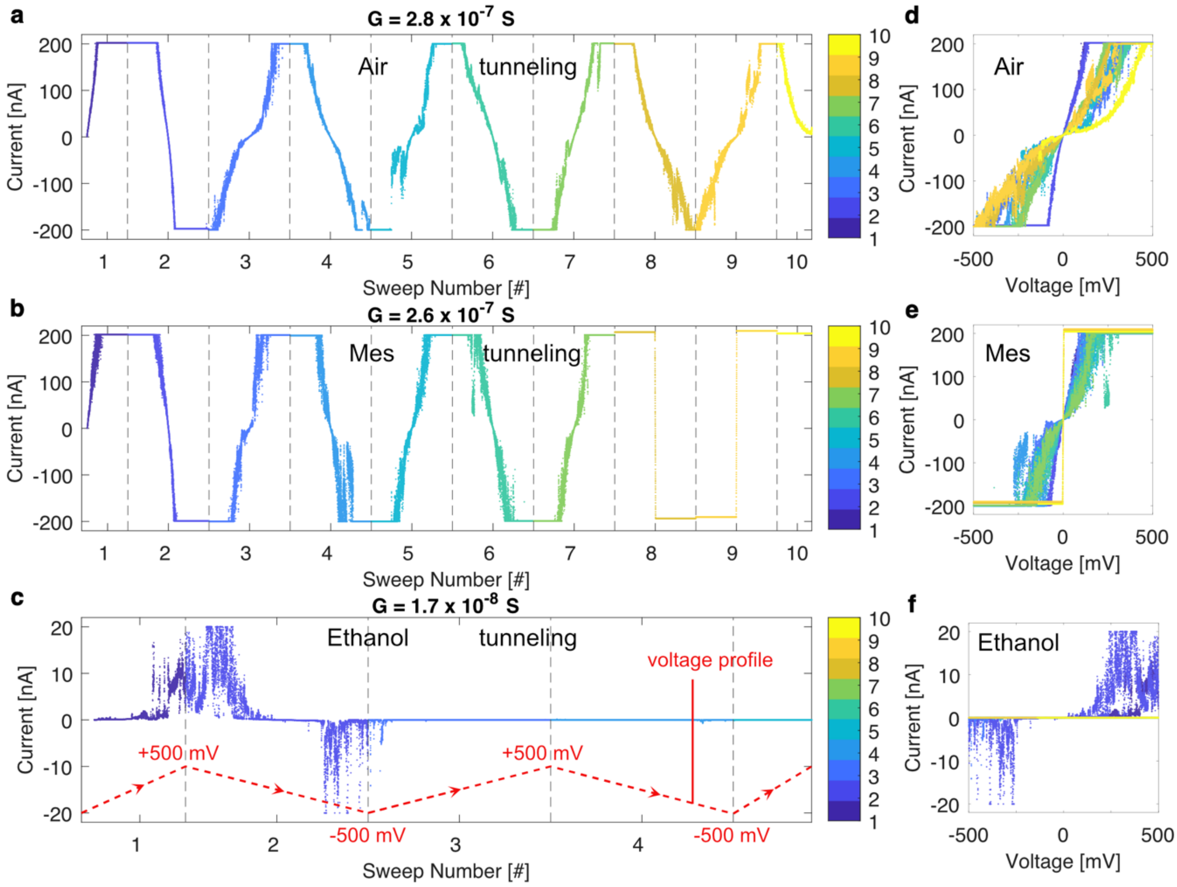

Figure S12. I-V characteristics of four devices showing reconfiguration in air, mesitylene (Mes) and ethanol. All sweeps have been colored by the sweep number shown in the colorbars. Two different representations of the same device is shown: (a)– (c) current vs sweep number to the left and (d) – (f) current vs voltage to the right. In panels a–c the x-axis is time but the axis is labeled by the the sweep number. A schematic of the voltage profile is shown in panel c and the veritcal dashed lines indicate where the voltage sweep direction between ±500 mV changes from increasing to decreasing and vice-versa. In air and mesitylene high conductance devices (> 100 nS) show abrupt changes in conductance at voltage bias of either polarity above ~ 250 mV. The devices typically remain responsive to applied voltage bias after reconfiguration. In ethanol all three devices which showed any response to voltage bias after self-breaking reconfigure and become low G devices after the application of a few I-V sweeps shown here.

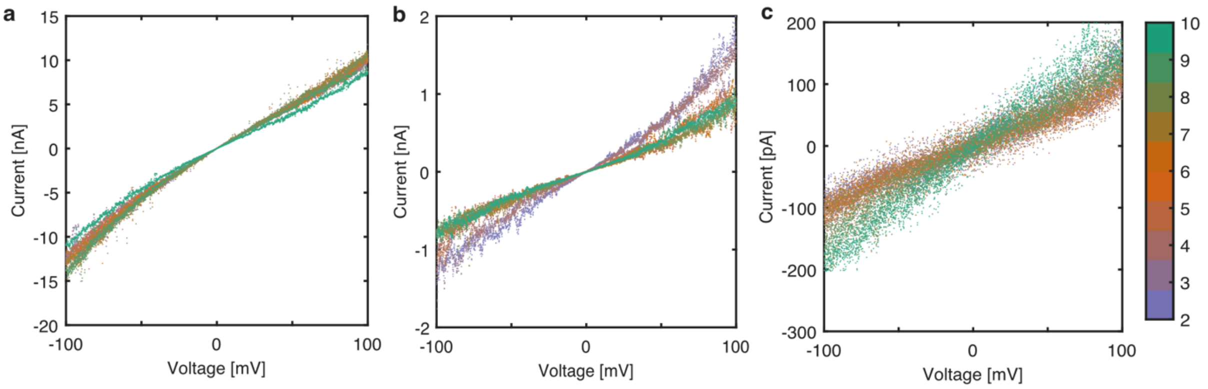

Figure S13. Low-level reconfiguration in mesitylene. I-V sweeps colored by ramp number for three devices (a)–(c) which showed reconfiguration in mesitylene even when only biased to maximum of 100 mV in the Mes-B* batch. Note however that the resulting conductance change is only small compared to the massive changes observed when biasing up to 500 mV (e.g., Fig 4h in the main text). The color bar applies to all figure panels.

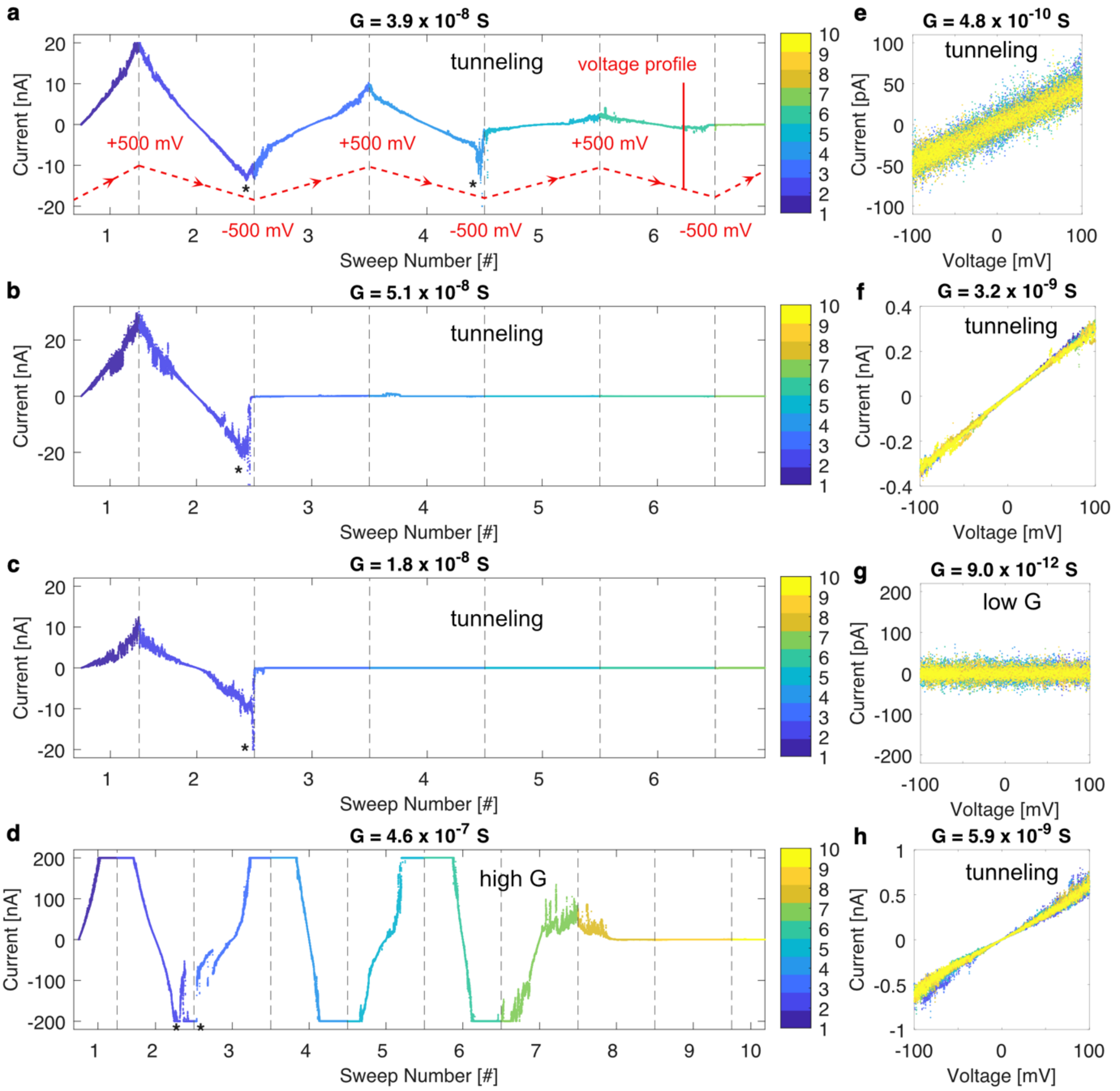

Figure S14. I-V characteristics of four devices showing reconfiguration in DIW. All sweeps have been colored by the sweep number shown in the colorbars. (a)-(c) I-V sweeps of tunneling devices to 500 mV where abrupt decreases in conductance was observed at negative voltage bias in one or more sweeps in DIW. The location of the initiation of such reconfiguration is indicated with an * in the plots. (d) A high G device showing a combination of reconfiguration observed in all media while applying high bias to highly conductive (>100 nS) devices, and the type of abrupt reconfiguration at negative bias observed in DIW. In panels a–d the veritcal dashed lines indicate where the voltage sweep direction changes from increasing to decreasing and vice-versa. (e)-(h) I-V sweeps of the same four devices to 100 mV taken ~12h later. Three of the four devices are tunneling and have much lower conductance than before reconfiguration but stable conductance characteristics. Note that the conductances (G) shown in the left panels for reconfiguring devices is computed from the first sweep only.

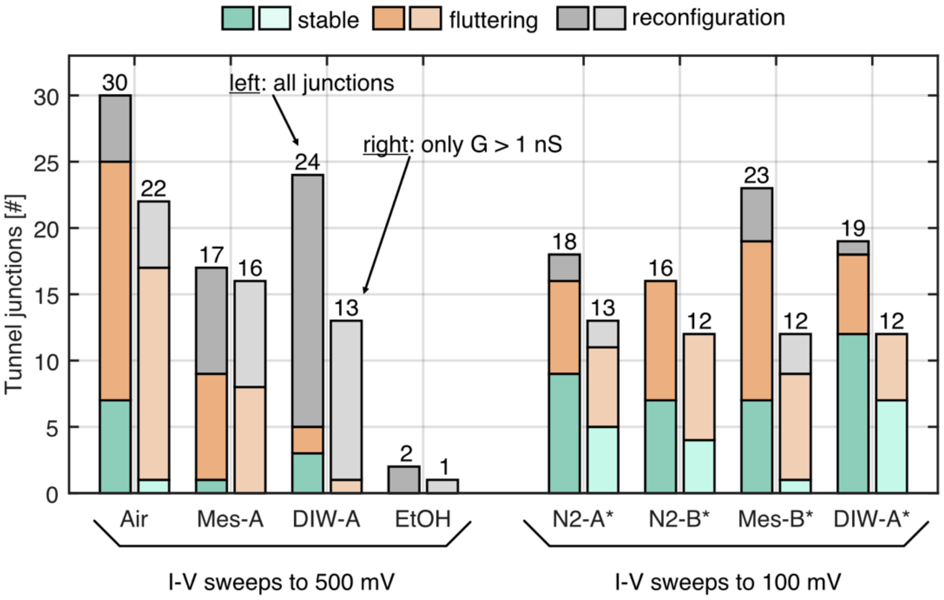

Figure S15. Distribution of conductance stability characteristics of tunnel junctions across all batches of measurements. Bar graphs showing the relative distribution of tunnel junctions categorized as stable, reconfiguration or fluttering, for all measurement batches. For each batch two bars are plotted: (left) all tunnel junctions; (right) only tunnel junctions with conductance (G) greater than 1 nS. The measurement batches measured to 500 mV during I-V sweeps show a greater incidence of significant reconfiguration and are grouped to the left. Among the measurement batches to the right, we notice that the devices in mesitylene (Mes-B*) show a significant tendency towards conductance instability when G > 1 nS compared to tunnel junctions in N_2 or DIW, as seen from the relative proportion of junctions which are stable. Note that the devices in DIW-A* are the same devices as in DIW-A. After the electrical stressing they were subjected to in DIW-A (where many devices reconfigured), a significant proportion of surviving devices show stable conductance characteristics in DIW-A*.

Supplementary Note 9: Parametric classification of the conductance stability of tunnel junctions from I-V characteristics.

For the following analysis a dataset containing all devices classified as tunneling from eight batches of measurements described in the manuscript (see Fig. 5) were considered. The aim was to identify a parameter from the I-V characteristics which was most effective at discriminating between stable and unstable devices. Visual inspection of the I-V characteristics (I-V histograms and I-V sweep colored by the sweep number, see Fig. 4) were used to assign stable or unstable labels to these devices. A total of 149 independent measurements were labeled in this way. After a thorough manual inspection, we established that devices exhibiting a standard deviation exceeding 10 nA at 0 V could be classified as unstable without further scrutiny, leading to a reduction of ten devices in our dataset size. All these devices were at the upper limit of conductance to be classified as tunneling and saturated the highest current range in our measurement (200 nA) at bias well below 100 mV.

Our analysis focused on a selection of features drawn from these histograms, emphasizing those exhibiting relatively high performance. As described in the Methods section of the manuscript, we define H as the matrix containing bin counts from the I-V histogram. $H_{i|v}$ signifies the bin count at current i and voltage v normalized by the total counts across all currents at voltage v. v_0 represents the voltage of zero volts. Finally, I stands for the set of all values of the current bins for a given device. We define the current range at a given voltage as

$$\mathrm{Range}_i(v) = i_\mathrm{M}(v) - i_\mathrm{m}(v),\ i_\mathrm{M}(v) = \max\{i \in I: H_{i|v} > 0\},\\ i_\mathrm{m}(v) = \min\{i \in I: H_{i|v} > 0\}. \tag{S1}$$

This measure can be seen as the difference between the minimum and the maximum current recorded for a certain voltage in the I-V histogram. Then using the marginal probabilities, the mean (μ_i) and variance (σ_i^2) of the current for a given voltage can be expressed as

$$\mu_i(v) \equiv \mathrm{Mean}_i(v) = \sum_{i\in I} i\, H_{i|v} \tag{S2}$$

$$\sigma_i^2(v) \equiv \mathrm{Var}_i(v) = \sum_{i\in I} (i - \mu_i(v))^2 H_{i|v} \tag{S3}$$

and the mean absolute error (ε_i) as

$$\varepsilon_i(v) \equiv \mathrm{MAE}_i(v) = \sum_{i\in I} |i - \mu_i(v)| H_{i|v} \tag{S4}$$

For each applied voltage bias, the mean current represents the device's signal in the absence of noise. We define the signal, denoted as S, as the highest absolute value among the current means, excluding instances where the device saturated. Noise, labeled as N, is defined as the fluctuations of the signal and can be characterized with its variance. In certain devices, the highest variance in currents did not occur at the highest/lowest voltage biases. Hence, we

identify the worst variance as the most representative indicator of noise for assessing device stability. Consequently, we define the signal-to-noise feature as:

$$SNR = \frac{1}{NSR} \equiv \frac{S^2}{E[N^2]} = \frac{\max_v \mu_i^2(v)}{\max_v \sigma_i^2(v)} \tag{S5}$$

Then the other analyzed features (F_R , F_A , F_V and F_{VC}) are defined as

$$F_R \equiv \log_{10}\left(max_v \frac{\text{Range}_i(v)}{\text{Range}_i(v_0)} \right) \tag{S6}$$

$$F_A \equiv \log_{10}\left(max_v \frac{\varepsilon_i(v)}{\varepsilon_i(v_0)} \right) \tag{S7}$$

$$F_V \equiv \log_{10}\left(max_v \frac{\sigma_i^2(v)}{\sigma_i^2(v_0)} \right) \tag{S8}$$

$$F_{VC} \equiv \log_{10}\left(max_v \frac{\sigma_i^2(v) - \sigma_i^2(v_0)}{\sigma_i^2(v_0)} \right) \tag{S9}$$

Each of these four features compares a metric maximized across all applied voltage biases, against a corresponding reference value. F_R assesses the range of occurrences without considering their relative frequency, comparing the worst one to the same metric at zero volts. Next, F_A employs absolute error as its comparison metric, while F_V utilizes variance. Lastly, F_{VC} incorporates device-related variance in the numerator, assuming independence from background noise, and divides it by the background noise.

Figure S16 presents a performance comparison of these features within the previously described dataset. For each feature, a straightforward threshold classification was employed. The feature values were arranged in order, and potential thresholds were considered as the midpoints between two neighbors' values of the feature. Accuracy was then assessed for each threshold, and the threshold that yielded the highest accuracy was selected.

The signal-to-noise ratio feature had an accuracy of 74.8 %, which is drastically lower than the other mentioned features, even though it improves classification based on random guess. While F_R exhibits an accuracy increase compared to the previous feature, it still falls short of the other features. Its limitation lies in its inability to account for current distributions at a given voltage, resulting in several misclassifications. In contrast, F_A considers the mentioned distribution and, consequently, achieves higher accuracy and better class separation. However, F_V surpasses even F_A in terms of accuracy and class separation. This improvement stems from the fact that variance captures fluctuations induced by noise and assigns greater weight to samples that deviate from the distribution. Lastly, F_{VC} isolates current fluctuations attributable to the device by considering the background noise as an independently added noise source. While this feature exhibits minimal improvements for highly unstable devices, it yields substantially lower values for stable devices compared to F_V. These value fluctuations enhance class separation, making F_{VC} the most effective feature for indicating stability within this analysis.

It's worth noting that we evaluated many more parameters but they exhibited inferior performance compared to the features discussed here. In conclusion, F_{VC} can be considered robust due to its effectiveness across various media. However, further data from diverse devices would be required to establish it as the definitive method for distinguishing stability with the greatest certainty.

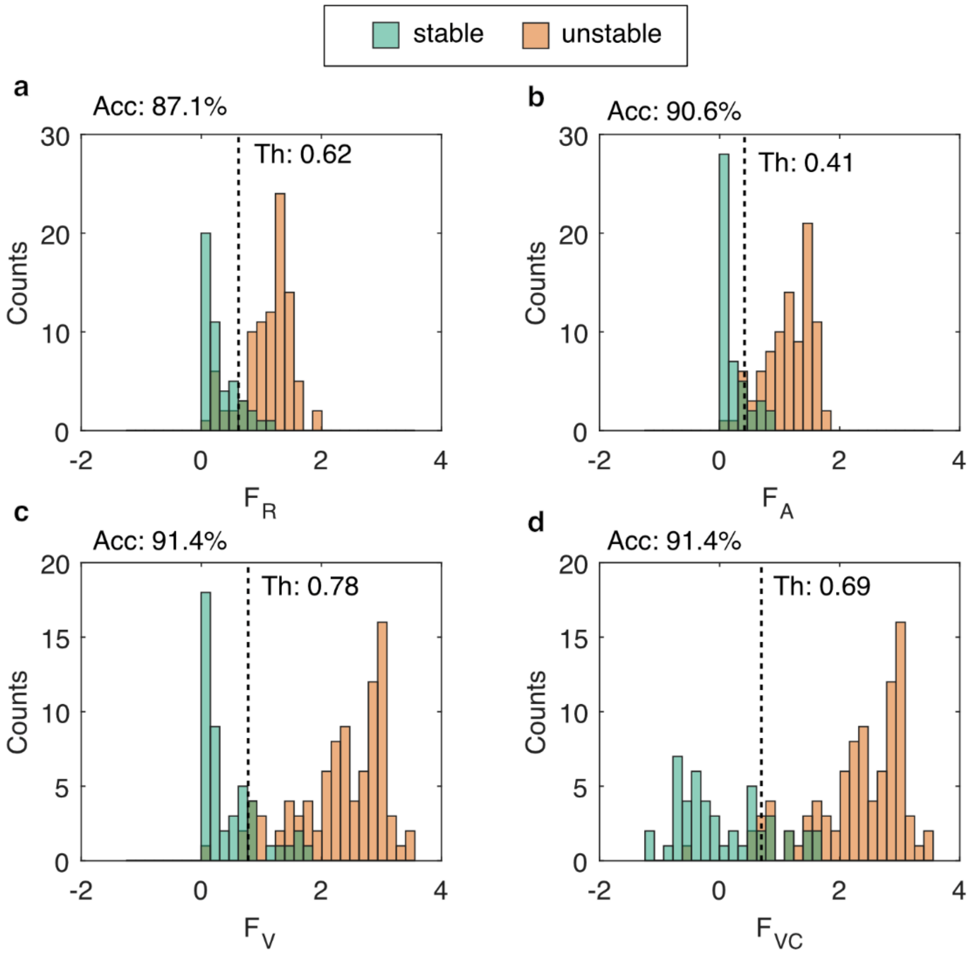

Figure S16. Comparison of different parameters analyzed to automate stability classification of tunnel junctions using their I-V characteristics. The histograms show the distribution of (a) F_R, (b) F_A, (c) F_V and (d) F_{VC} values of devices classified manually as stable or unstable. In each case a single threshold value for the respective parameter can be identified which achieves the highest accuracy to parametrically classify any individual nanogap tunnel junction as stable if it lies to the left of the threshold, and as unstable if it lies to the right of the threshold. The threshold value (Th) for each parameter and the accuracy (Acc) of classification achieved in each case is shown in the respective figure panel. We observe the highest accuracy and the broadest separation between stable and unstable devices while using F_{VC} as the parameter for classification, as seen by comparing (d) to (a)–(c).

Supplementary Note 10. Charts summarizing the procedure and key findings of measurement series in mesitylene.

48 devices were measured three times in batches to determine the stability of conductance characteristics. Taking the start time of measurement of Batch 1 as the time origin, t = 0h, Batch 2 and 3 were measured at t = 3h and t = 22h respectively. The mesitylene perfused chip was left mounted in the setup till all measurements were complete.

Procedure summary:

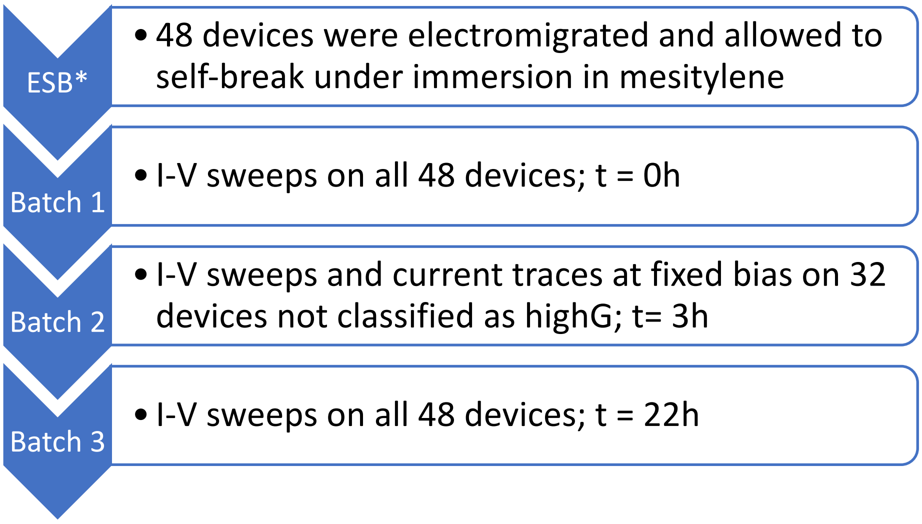

*** ESB : Electromigration and self-breaking**

Results summary:

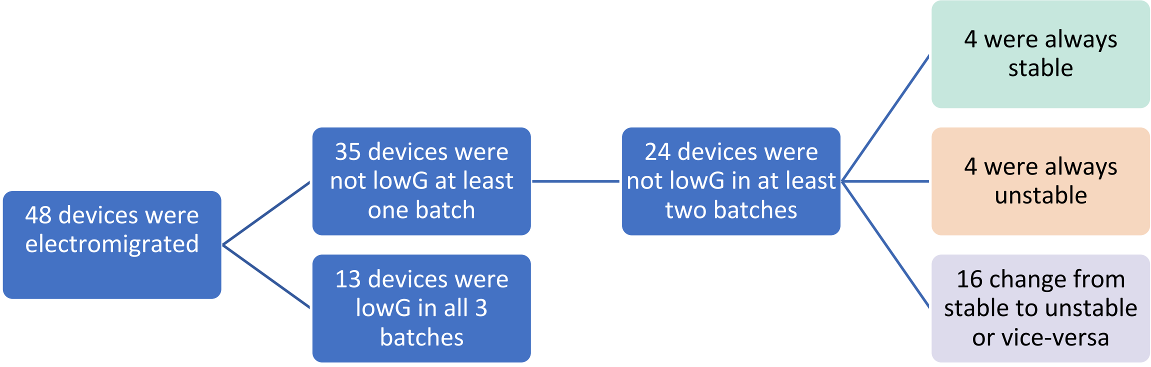

Supplementary References

(1) van der Zant, H. S. J.; Osorio, E. A.; Poot, M.; O'Neill, K. Electromigrated Molecular Junctions. *physica status solidi (b)* **2006**, *243* (13), 3408–3412. https://doi.org/10.1002/pssb.200669185.

(2) O'Neill, K.; Osorio, E. A.; van der Zant, H. S. J. Self-Breaking in Planar Few-Atom Au Constrictions for Nanometer-Spaced Electrodes. *Appl Phys Lett* **2007**, *90* (13), 133109. https://doi.org/10.1063/1.2716989.

(3) Hoffmann-Vogel, R. Electromigration and the Structure of Metallic Nanocontacts. *Appl Phys Rev* **2017**, *4* (3), 031302. https://doi.org/10.1063/1.4994691.

(4) Morikawa, T.; Yokota, K.; Tsutsui, M.; Taniguchi, M. Fast and Low-Noise Tunnelling Current Measurements for Single-Molecule Detection in an Electrolyte Solution Using Insulator-Protected Nanoelectrodes. *Nanoscale* **2017**, *9* (12), 4076–4081. https://doi.org/10.1039/C6NR09278K.

(5) Ward, D. R.; Grady, N. K.; Levin, C. S.; Halas, N. J.; Wu, Y.; Nordlander, P.; Natelson, D. Electromigrated Nanoscale Gaps for Surface-Enhanced Raman Spectroscopy. *Nano Lett* **2007**, *7* (5), 1396–1400. https://doi.org/10.1021/nl070625w.

(6) Tsutsui, M.; Rahong, S.; Iizumi, Y.; Okazaki, T.; Taniguchi, M.; Kawai, T. Single-Molecule Sensing Electrode Embedded in-Plane Nanopore. *Sci Rep* **2011**, *1* (1), 46. https://doi.org/10.1038/srep00046.

(7) Suga, H.; Suzuki, H.; Otsu, K.; Abe, T.; Umeta, Y.; Tsukagoshi, K.; Sumiya, T.; Shima, H.; Akinaga, H.; Naitoh, Y. Feedback Electromigration Assisted by Alternative Voltage Operation for the Fabrication of Facet-Edge Nanogap Electrodes. *ACS Appl Nano Mater* **2020**, *3* (5), 4077–4083. https://doi.org/10.1021/acsanm.0c00138.

(8) Frimmer, M.; Puebla-Hellmann, G.; Wallraff, A.; Novotny, L. The Role of Titanium in Electromigrated Tunnel Junctions. *Appl Phys Lett* **2014**, *105* (22), 221118. https://doi.org/10.1063/1.4903748.

(9) Todeschini, M.; Bastos da Silva Fanta, A.; Jensen, F.; Wagner, J. B.; Han, A. Influence of Ti and Cr Adhesion Layers on Ultrathin Au Films. *ACS Appl Mater Interfaces* **2017**, *9* (42), 37374–37385. https://doi.org/10.1021/acsami.7b10136.

(10) Kozlova, T.; Rudneva, M.; Zandbergen, H. W. *In Situ* TEM and STEM Studies of Reversible Electromigration in Thin Palladium–Platinum Bridges. *Nanotechnology* **2013**, *24* (50), 505708. https://doi.org/10.1088/0957-4484/24/50/505708.

(11) Strachan, D. R.; Smith, D. E.; Johnston, D. E.; Park, T.-H.; Therien, M. J.; Bonnell, D. A.; Johnson, A. T. Controlled Fabrication of Nanogaps in Ambient Environment for Molecular Electronics. *Appl Phys Lett* **2005**, *86* (4), 043109. https://doi.org/10.1063/1.1857095.

(12) Strachan, D. R.; Smith, D. E.; Fischbein, M. D.; Johnston, D. E.; Guiton, B. S.; Drndić, M.; Bonnell, D. A.; Johnson, A. T. Clean Electromigrated Nanogaps Imaged by Transmission Electron Microscopy. *Nano Lett* **2006**, *6* (3), 441–444. https://doi.org/10.1021/nl052302a.

(13) Stöffler, D.; Fostner, S.; Grütter, P.; Hoffmann-Vogel, R. Scanning Probe Microscopy Imaging of Metallic Nanocontacts. *Phys Rev B Condens Matter Mater Phys* **2012**, *85* (3). https://doi.org/10.1103/PhysRevB.85.033404.

(14) Grüter, L.; González, M. T.; Huber, R.; Calame, M.; Schönenberger, C. Electrical Conductance of Atomic Contacts in Liquid Environments. *Small* **2005**, *1* (11), 1067–1070. https://doi.org/10.1002/smll.200500145.

(15) Ivanov, A. P.; Freedman, K. J.; Kim, M. J.; Albrecht, T.; Edel, J. B. High Precision Fabrication and Positioning of Nanoelectrodes in a Nanopore. *ACS Nano* **2014**, *8* (2), 1940–1948. https://doi.org/10.1021/nn406586m.

(16) Tang, L.; Nadappuram, B. P.; Cadinu, P.; Zhao, Z.; Xue, L.; Yi, L.; Ren, R.; Wang, J.; Ivanov, A. P.; Edel, J. B. Combined Quantum Tunnelling and Dielectrophoretic Trapping for Molecular Analysis at Ultra-Low Analyte Concentrations. *Nat Commun* **2021**, *12* (1), 1–8. https://doi.org/10.1038/s41467-021-21101-x.

(17) Prokopuk, N.; Son, K.; Waltz, C. Electron Tunneling through Fluid Solvents. *The Journal of Physical Chemistry C* **2007**, *111* (17), 6533–6537. https://doi.org/10.1021/jp070106h.

(18) Mangin, A.; Anthore, A.; Della Rocca, M. L.; Boulat, E.; Lafarge, P. Reduced Work Functions in Gold Electromigrated Nanogaps. *Phys Rev B* **2009**, *80* (23), 235432. https://doi.org/10.1103/PhysRevB.80.235432.

(19) Kießig, B.; Schäfer, R.; von Löhneysen, H. Two-Dimensional Simulations of Temperature and Current-Density Distribution in Electromigrated Structures. *New J Phys* **2014**, *16* (1), 013017. https://doi.org/10.1088/1367-2630/16/1/013017.
